# A structured weight loss program increases gut microbiota phylogenetic diversity and reduces levels of *Collinsella* in obese type 2 diabetics: A pilot study

**DOI:** 10.1371/journal.pone.0219489

**Published:** 2019-07-18

**Authors:** Fabian Frost, Lena J. Storck, Tim Kacprowski, Simone Gärtner, Malte Rühlemann, Corinna Bang, Andre Franke, Uwe Völker, Ali A. Aghdassi, Antje Steveling, Julia Mayerle, Frank U. Weiss, Georg Homuth, Markus M. Lerch

**Affiliations:** 1 Department of Medicine A, University Medicine Greifswald, Greifswald, Germany; 2 Department of Medicine, Cantonal Hospital Winterthur, Winterthur, Switzerland; 3 Department of Functional Genomics, Interfaculty Institute for Genetics and Functional Genomics, University Medicine Greifswald, Greifswald, Germany; 4 Chair of Experimental Bioinformatics, TUM School of Life Sciences Weihenstephan, Technical University of Munich, Freising-Weihenstephan, Germany; 5 Institute of Clinical Molecular Biology, Christian Albrechts University of Kiel, Kiel, Germany; 6 Department of Medicine II, University Hospital, LMU Munich, Munich, Germany; Weill Cornell Medical College in Qatar, QATAR

## Abstract

The global obesity epidemic constitutes a major cause of morbidity and mortality challenging public health care systems worldwide. Thus, a better understanding of its pathophysiology and the development of novel therapeutic options are urgently needed. Recently, alterations of the intestinal microbiome in the obese have been discussed as a promoting factor in the pathophysiology of obesity and as a contributing factor to related diseases such as type 2 diabetes and metabolic syndrome. The present pilot study investigated the effect of a structured weight loss program on fecal microbiota in obese type 2 diabetics. Twelve study subjects received a low-calorie formula diet for six weeks, followed by a nine week food reintroduction and stabilization period. Fecal microbiota were determined by 16S rRNA gene sequencing of stool samples at baseline, after six weeks and at the end of the study after fifteen weeks. All study subjects lost weight continuously throughout the program. Changes in fecal microbiota were most pronounced after six weeks of low-calorie formula diet, but reverted partially until the end of the study. However, the gut microbiota phylogenetic diversity increased persistently. The abundance of *Collinsella*, which has previously been associated with atherosclerosis, decreased significantly during the weight loss program. This study underlines the impact of dietary changes on the intestinal microbiome and further demonstrates the beneficial effects of weight loss on gut microbiota.

**Trial registration:** ClinicalTrials.gov NCT02970838.

## Introduction

The world is stricken by an obesity epidemic that affects not only over 32% of the population within the US [1], but is also becoming increasingly prevalent in developing countries [2]. This is accompanied by a sharp rise of type 2 diabetes leading to growing numbers of cardiovascular disease cases, increasing morbidity and mortality [3]. The pathogenesis of obesity and type 2 diabetes are characterized by multiple factors including a high calorie diet, a sedentary lifestyle, and genetics [3, 4]. First-line treatment focuses on dietary interventions and increased physical activity. If these measures fail, medication and, with increasing frequency, bariatric surgery are further possible treatment options. The success of these therapies varies considerably between individuals and the underlying factors that determine success or failure of the chosen interventions are incompletely understood. For a better characterization of the pathogenesis and therapy of obesity as well as type 2 diabetes, scientists have recently focused on the intestinal microbiome. This has become feasible due to the emergence of next generation sequencing techniques, which have overcome the shortcomings of culturing techniques in the characterization of the mainly anaerobic gut bacteria. It was shown that intestinal microbiota play a role in the breakdown of otherwise indigestible dietary components such as plant polysaccharides [5] and further animal experiments revealed that obesity is associated with a gut microbiome of increased capability for energy harvest [6]. Obesity has even been shown to be transmissible by transferring the intestinal microbiome between mice. Germ-free recipients of obese donors gained significantly more body fat than germ-free recipients of lean donors [6]. Likewise in humans, transferring the intestinal microbiome of lean donors to individuals suffering from metabolic syndrome increased the insulin sensitivity of the recipients [7]. Other reported characteristics of intestinal microbiota in the obese include a reduction of the microbial diversity [8, 9] and an increased *Firmicutes/Bacteroidetes* ratio [10]. Yet, the latter could not be replicated consistently and remains controversial [11, 12]. In individuals suffering from type 2 diabetes, presence of moderate dysbiosis with an increase in facultative pathogenic microbes has also been demonstrated [13] and the degree of glucose intolerance associated with specific shifts in the gut microbiota composition in another study [14]. The intestinal microbiome can be modified by dietary interventions and/or weight loss. Although several weight loss studies have targeted the microbiome in the obese and/or type 2 diabetics, the results are heterogeneous and sometimes contradictory [10, 15–18]. To further characterize the effect of dietary interventions and weight loss on the intestinal microbiome, we performed a pilot study investigating fecal microbiota characterized by 16S rRNA gene sequencing in twelve obese type 2 diabetics at several time points during a structured weight loss program.

## Materials and methods

### Study subjects

**Fig 1:**
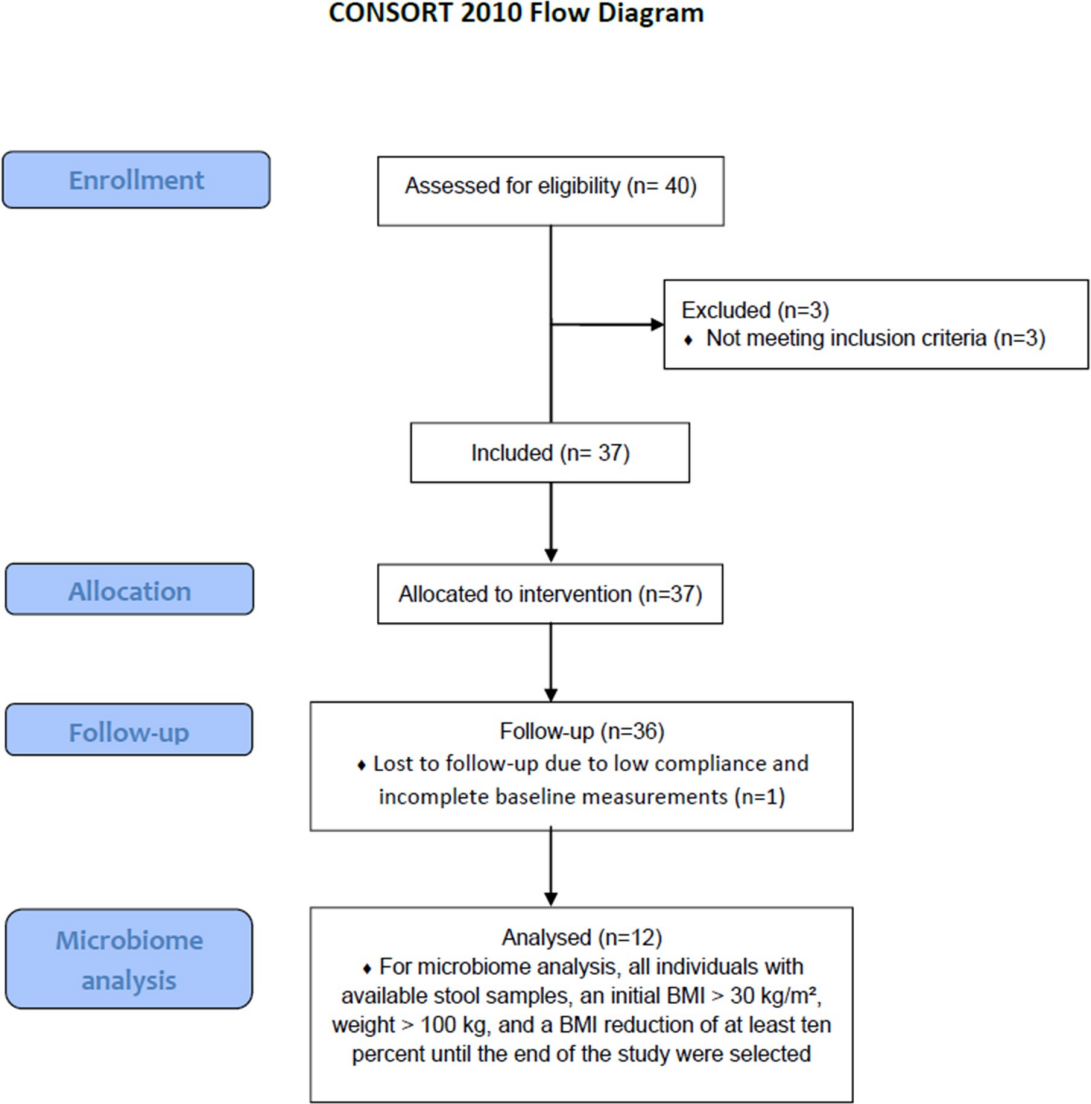
CONSORT flow diagram.

All data analyzed within the present work were obtained from the TADIA (Therapy of Obesity and Diabetes Mellitus Type 2) study which included obese type 2 diabetics who participated in a standardized weight loss program [19]. As a potential effect size of the weight loss program on intestinal microbiota composition was unknown, no a priory power estimate regarding sample size for microbiota changes was possible. The study protocol that accompanies the present manuscript relates to the TADIA study which was approved by the local ethics committee of Greifswald University Hospital, Germany (No. BB062/12, 30/05/2012) and registered at ClinicalTrials.gov (NCT02970838). The authors confirm that all ongoing and related trials for this intervention are registered. For recruitment of participants (**Fig 1** shows CONSORT flow diagram), advertisements were placed in selected local newspapers and interested individuals had to call the investigators on a central telephone number. At the time of inclusion, participants had to be known type 2 diabetics, aged between 18 and 70 years, with a body mass index (BMI) of at least 27 kg/m^2^. Exclusion criteria for participation were current treatment with incretin mimetic drugs within the last three months, pregnancy, immobilization, severe heart, liver or renal failure, allergy to the formula diet, dementia, eating disorders, or alcoholism. Participant recruitment was performed in the period from 05/11/2012 to 28/01/2014. The last follow-up examination was performed on the 06/05/2014. The initial cohort comprised 36 individuals. The collection of stool samples was only initiated after 8 individuals had already completed the program. Two further participants did not provide stool samples at all time points. Eventually, 26 individuals collected stool samples at baseline, six, and fifteen weeks. For the present study we selected all individuals with available stool samples, an initial BMI of > 30 kg/m^2^, an initial weight of > 100 kg, and a BMI reduction of at least ten percent until the end of the study. Of the twelve selected participants, eight were of female and four of male gender. The mean age was 57.2 years. Treatment of diabetes was performed dietetically in five, with oral medication in four, and with combined oral and insulin medication in three cases. Eleven individuals were suffering from hypertension and eight participants had hyperlipidemia of which seven were receiving antihyperlipidemic agents. One study subject was a smoker. All study participants provided written informed consent before inclusion in the trial.

### Standardized weight loss program

All participants received a standardized three-phase weight loss program (OPTIFAST II Short program, Nestlé Health Science Germany). The initial fasting phase lasted six weeks during which individuals received a low-calorie formula diet fully substituting the regular diet. Five formula sachets containing 96 g of carbohydrates, 70 g of proteins and 15 g of fat were consumed per day providing 800 kcal energy. In addition, the sachets were enriched with the daily recommended amount of minerals and vitamins. Apart from the formula diet, patients were recommended to drink at least 2.5 liters of water or any other calorie-free beverage per day. During the second phase, comprising four weeks, the formula diet was incrementally reduced and a regular diet reintroduced, allowing a daily intake of 1,200 kcal. The final phase lasted five weeks wherein the daily energy intake was incrementally increased up to the predicted amount that would stabilize the participants weight (1,200–1,500 kcal). In addition to dietary instructions, all participants received a structured physical training course adapted to the individual fitness level throughout the intervention. Health status and diabetic drug dosage were monitored once a week by the study center. To avoid the occurrence of hypoglycemic episodes, oral insulinotropic drugs and metformin were paused during the fasting phase. Furthermore, individuals requiring prandial insulin injections received a lowered dose of two insulin units per carbohydrate unit. All subjects were advised to monitor their blood glucose levels at least six times daily and to inform the study physician in case of levels below 5.6 mmol/l or higher than 12 mmol/l. Further adjustments of insulin dosages were performed on an individual basis when necessary.

### Phenotypic data

Body mass index (BMI) was calculated as kilograms per square meter. Blood for determination of HbA1c, fasting glucose, insulin, total cholesterol, low density lipoprotein (LDL), high density lipoprotein (HDL), triglycerides, and uric acid was obtained in the morning after a minimum fasting period of eight hours.

### 16S rRNA gene sequencing

All fecal samples were collected in the domestic environment at baseline, six and fifteen weeks by the study participants, transferred into a stabilizing EDTA-buffer and then shipped to our laboratory. DNA extraction was done using the PSP Spin Stool DNA Kit (Stratec Biomedical AG, Birkenfeld, Germany) and the isolated DNA stored at -80°C. The V1-V2 region of bacterial 16S rRNA genes was amplified by dual-indexing PCR using the primer pair 27F and 338R. The resulting PCR products were purified and normalized utilizing the Invitrogen SequalPrep Normalization Plate Kit (Thermo Fisher Scientific, Waltham, USA). Sequencing was performed on a MiSeq platform (Illumina, San Diego, USA). MiSeq Fast-Q files were generated by CASAVA 1.8.2 (https://support.illumina.com/sequencing/sequencing_software/casava) and all sequences trimmed with Sickle (https://github.com/najoshi/sickle). For merging of forward and reverse reads and subsequent filtering VSEARCH [20] was used. Quality filtering was performed by FastX Toolkit (http://hannonlab.cshl.edu/fastx_toolkit), including only reads with a quality score of at least 30 (error probability 1 in 1,000) per base in 95% of sequenced nucleotides. Clustering of operational taxonomic units (OTU) was done at a minimum sequence similarity of 97% using VSEARCH. Possible chimera were filtered out by USEARCH [21]. After randomly selecting 10,000 reads from each sample, taxonomic assignment was carried out using the RDP classifier at a minimum bootstrap confidence of 80% at each taxonomic rank. Classifications with confidence below 80% were assigned to an artificial taxon as unclassified family, order, class, or phylum.

### Outcomes

Fecal microbiota composition based on 16S rRNA gene sequencing.

### Data and statistical analysis

All statistical analyses and creation of figures were performed in 'R' (v. 3.3.3, *https*:*//www*.*r-project*.*org/*). Alpha diversity estimations based on OTU counts were calculated using the R package 'vegan' [22] for 'Simpson diversity number' and 'picante' [23] for 'Phylogenetic diversity'. Calculation of *Bray-Curtis dissimilarity* index and subsequent principal coordinate analysis (PCoA) was performed using the 'vegan' functions *vegdist* and *cmdscale*, respectively, on square root transformed genus abundance data. The significance of changes in beta diversity during the study was estimated using the 'vegan' function *adonis*, which fits linear models to distance matrices and calculates p values by permutations (n = 10,000). Paired continuous phenotypic variables and laboratory parameters were assessed with the Wilcoxon signed rank test (WT) or the Friedman test (FT) in case of two or three groups for comparison, respectively. To evaluate differences in fecal microbiota all phyla or genera that were present in more than 50% of samples were compared using the Friedman test and the resulting p values corrected for multiple testing by the procedure of Benjamini & Hochberg. Thereafter, the corrected p values were called q values. To further determine the specific time point at which taxa identified as significant by the global Friedman test were changed, taxon abundances at baseline were compared to six or fifteen weeks, respectively, using the Wilcoxon-signed rank test. All p or q values are given with three significant digits. Results were considered significant when q values were < 0.05 in case of taxon comparisons at different time points. For all other statistical analyses a p value < 0.05 was considered significant. All binary variables are stated as percentages and continuous variables as medians (first to third quartiles).

## Results

Twelve obese study subjects suffering from type 2 diabetes underwent a structured weight loss program which consisted of a low-calorie formula diet for 6 weeks and a subsequent food reintroduction and stabilization period for another 9 weeks. In addition, all participants performed structured physical exercises throughout the program. The aim was to investigate the effect of the weight loss program on fecal microbiota. Stool samples were collected at baseline, six and fifteen weeks and the microbial community structure determined by 16S rRNA gene sequencing.

Throughout the program, all participants lost weight continuously, starting at a BMI of 39.6 (36.6–40.9) kg/m^2^, decreasing to 35.4 (32.5–36.2) kg/m^2^ at six weeks and finally reaching 33.1 (30.1–34.2) kg/m^2^ after fifteen weeks (p < 0.001, FT). The absolute weight loss in all participants over the whole program ranged from 11.4 to 30.1 kg. This was accompanied by a significant improvement of glucose metabolism indicated by a reduction of HbA1c, fasting glucose, and insulin (**Table 1 and S1 Fig**). Furthermore, a reduction of total cholesterol and uric acid, which was most pronounced at the end of the fasting phase after six weeks, was noted.

**Table 1:** Laboratory evaluation of metabolic parameters at different study time points.

	Baseline (0 weeks)	6 weeks	15 weeks	p value
HbA1c (%)	6.6 (6.1–7.2)	-	6.0 (5.4–6.4)	0.003*
Fasting glucose (mmol/l)^#^	6.9 (6.2–8.3)	-	5.8 (5.4–6.2)	0.002*
Insulin (pmol/l)^#^	154.3 (102.4–217.8)	-	84.3 (53.1–99.8)	0.002*
Total cholesterol (mmol/l)^#^	4.7 (4.5–5.8)	4.5 (3.2–4.8)	4.8 (4.0–4.8)	0.016*
LDL (mmol/l)^#^	2.7 (2.6–3.4)	2.7 (1.9–3.2)	2.9 (2.6–3.0)	0.035*
HDL (mmol/l)^#^	1.2 (0.9–1.4)	1.0 (1.0–1.3)	1.1 (1.0–1.3)	0.234
Triglycerides (mmol/l)^#^	1.6 (1.1–2.5)	1.0 (0.9–1.6)	1.5 (0.9–2.2)	0.178
Uric acid (μmol/l)	316.0 (282.8–356.2)	250.5 (200.8–299.5)	273.5 (249.5–354.2)	0.024*

Metabolic laboratory parameters of the twelve participants were evaluated at baseline (0 weeks), after the low-calorie formula diet (6 weeks) and after reintroduction of a regular diet (15 weeks). LDL: Low density lipoprotein. HDL: High density lipoprotein. Data are given as median (first to third quartiles). Wilcoxon signed-rank test and Friedman test were used for assessment of significance in case of two or three different time points, respectively.

^#^ one participant was not fasting before the blood sample was obtained and therefore excluded from the analysis.

* indicates significant test result. All values were rounded to one decimal place.

### Weight loss is associated with increased phylogenetic diversity

**Fig 2:**
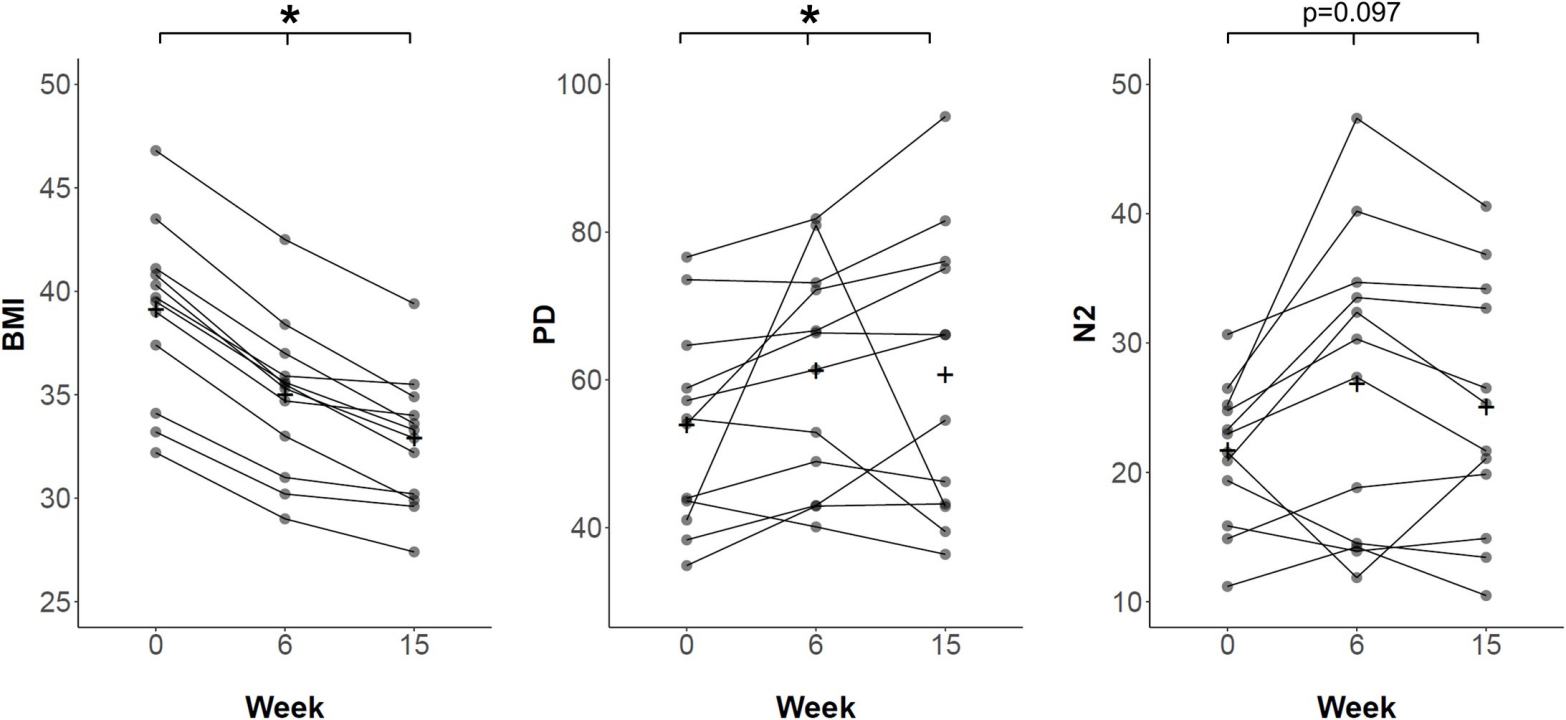
Development of body mass index and alpha diversity indices over the study course. Plots are portraying the changes of body mass index (BMI, left), Phylogenetic diversity (PD, middle) and Simpson diversity number (N2, right) at baseline/0 weeks, after six weeks of low-calorie formula diet and after regular food reintroduction at fifteen weeks. At each time point paired samples of 12 participants were evaluated. Samples corresponding to one individual are connected by a black line. + signs indicate the means. The decrease of BMI during the study was associated with an increase of PD. * indicates significant difference determined by Friedman test.

Alpha diversity indices describe the species variation within a community or sample. To assess the effect of weight loss on the gut microbiota alpha diversity, two different metrics, namely 'Phylogenetic diversity' (PD)[24] and 'Simpson diversity number' (N2)[25], were calculated and compared to baseline (**Fig 2**). PD, which is based on the phylogenetic relationship between different taxa within a sample, increased significantly from 54.4 (43.0–60.3) at baseline to 63.9 (47.5–72.4) after six weeks and 60.3 (43.1–75.3) after fifteen weeks (global: p = 0.039, FT; 0 vs. 6 weeks: p = 0.009, post-hoc WT; 0 vs. 15 weeks: p = 0.034, post-hoc WT). N2, which is based on taxon abundance data and diversity, was 22.2 (18.5–24.9) at baseline and increased to 28.8 (14.4–33.8) after six weeks and 23.5 (18.6–33.1) after fifteen weeks. However, the latter was not significant (p = 0.097, FT).

### Beta diversity analysis reveals distinct fecal microbiota shifts during the study

**Fig 3:**
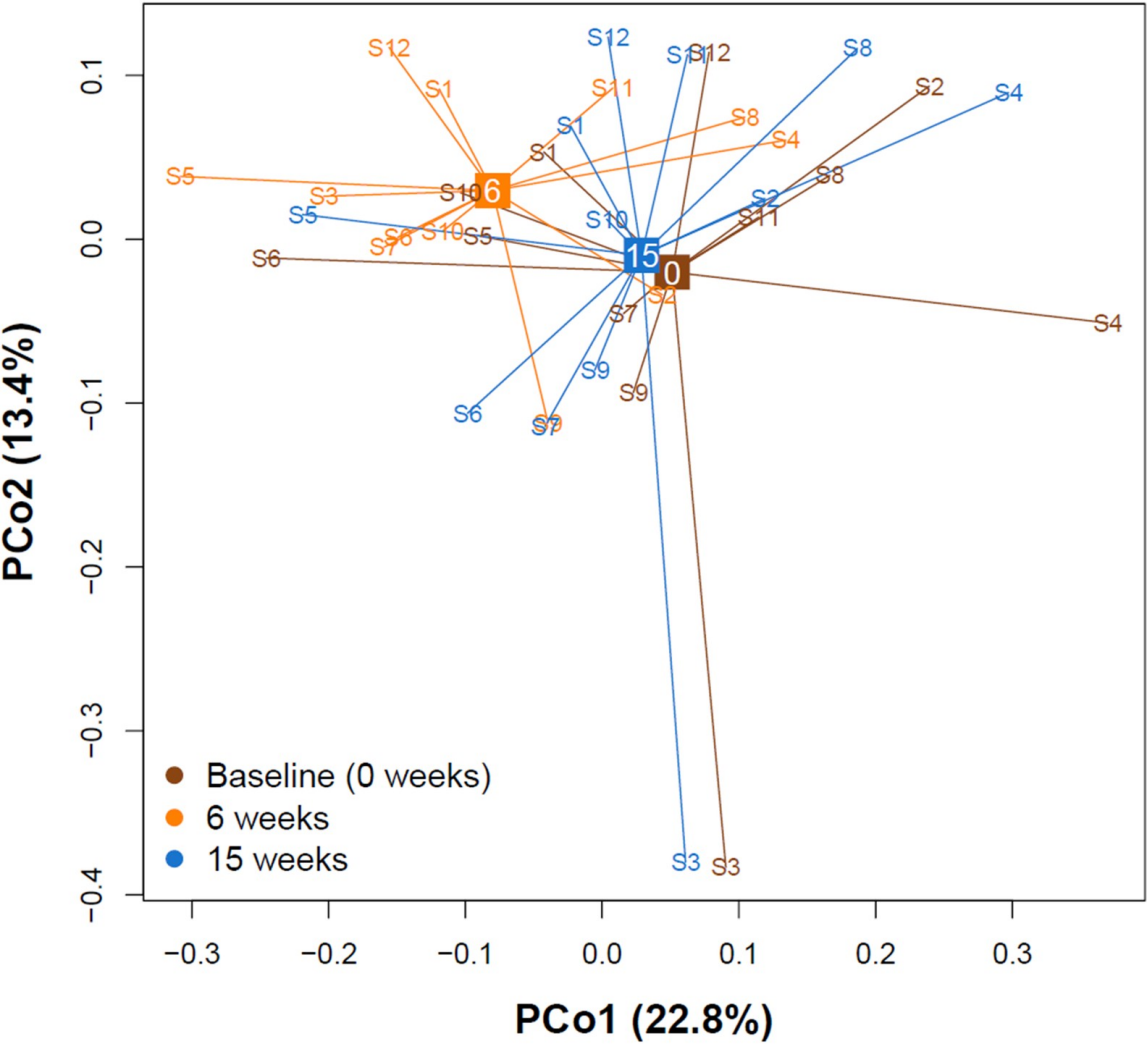
Principal coordinate analysis (PCoA) of Bray-Curtis dissimilarity. PCoA of 36 gut microbiota samples belonging to twelve participants at three different time points (baseline/0 weeks, six weeks and fifteen weeks). The two major PCo1 and PCo2 are shown. All samples are connected to the centroid (displayed as square) of their respective time point. The microbial communities shifted markedly between baseline and six weeks, then returned towards baseline after fifteen weeks.

Beta diversity indices assess the variation in composition between different communities or samples. To ascertain whether differences between gut microbiota at different time points were detectable, we computed the *Bray-Curtis dissimilarity*[25] and subsequently conducted a principal coordinate analysis. **Fig 3** shows the first two principal coordinate axes explaining 36.2% of the total microbial variation. The fecal microbiota markedly shifted after the end of the low-calorie formula diet (six weeks). Yet, at the end of the study (fifteen weeks) they returned towards baseline again. Performing permutational analysis of variance using *Bray-Curtis dissimilarity* confirmed significant shifts in microbiota diversity between the different time points (r^2^ = 0.08, p < 0.001).

### The majority of taxon abundance changes are not sustained after termination of the low-calorie formula diet

**Fig 4:**
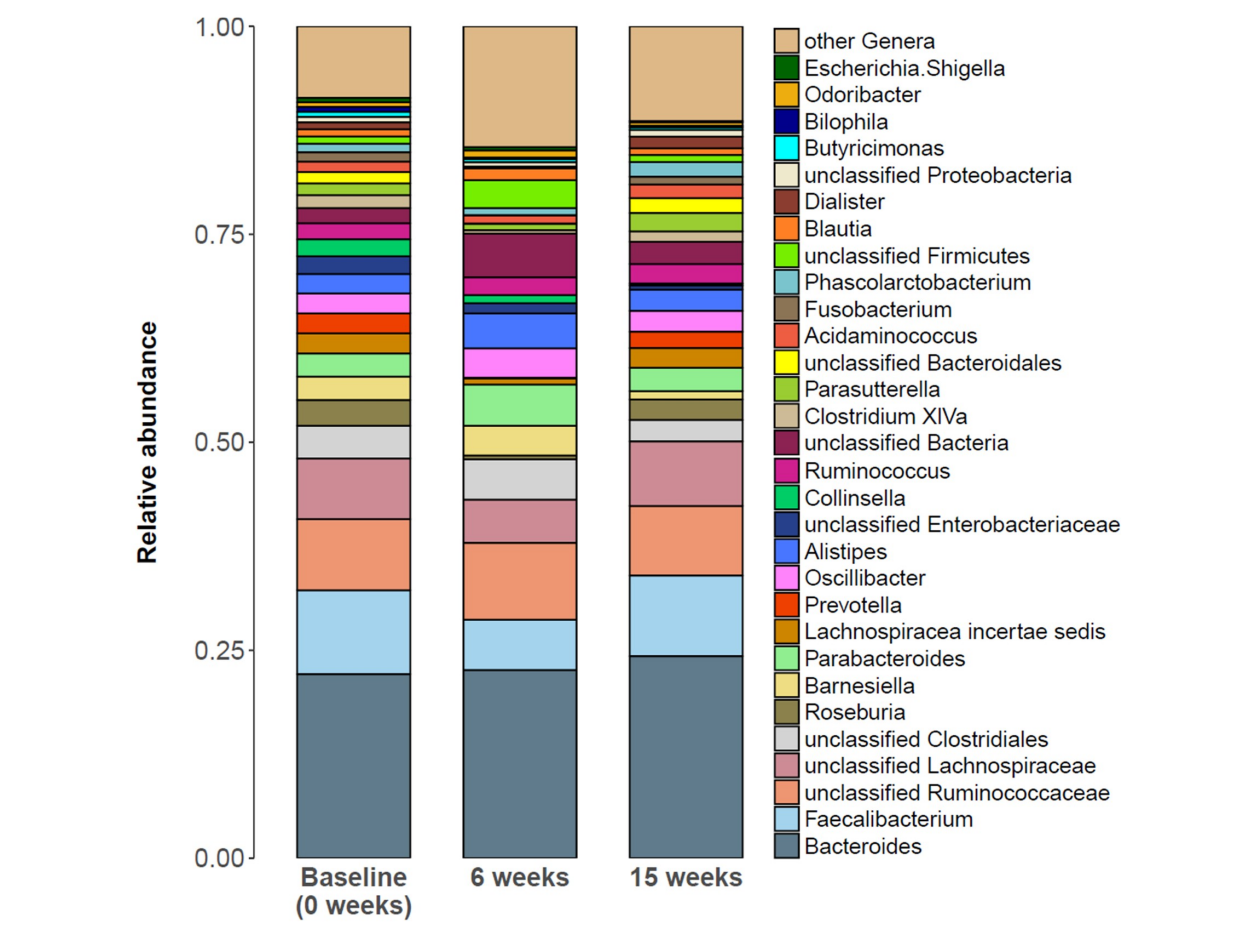
Taxon composition at baseline, 6 and 15 weeks after study launch. Stacked bar plots portraying the average sample composition of the study group (n = 12) at baseline/0 weeks, after six weeks of low-calorie formula diet and after regular food reintroduction at fifteen weeks. Taxon abundance changes were most prominent at six weeks, but mostly not sustained at fifteen weeks.

**Fig 5:**
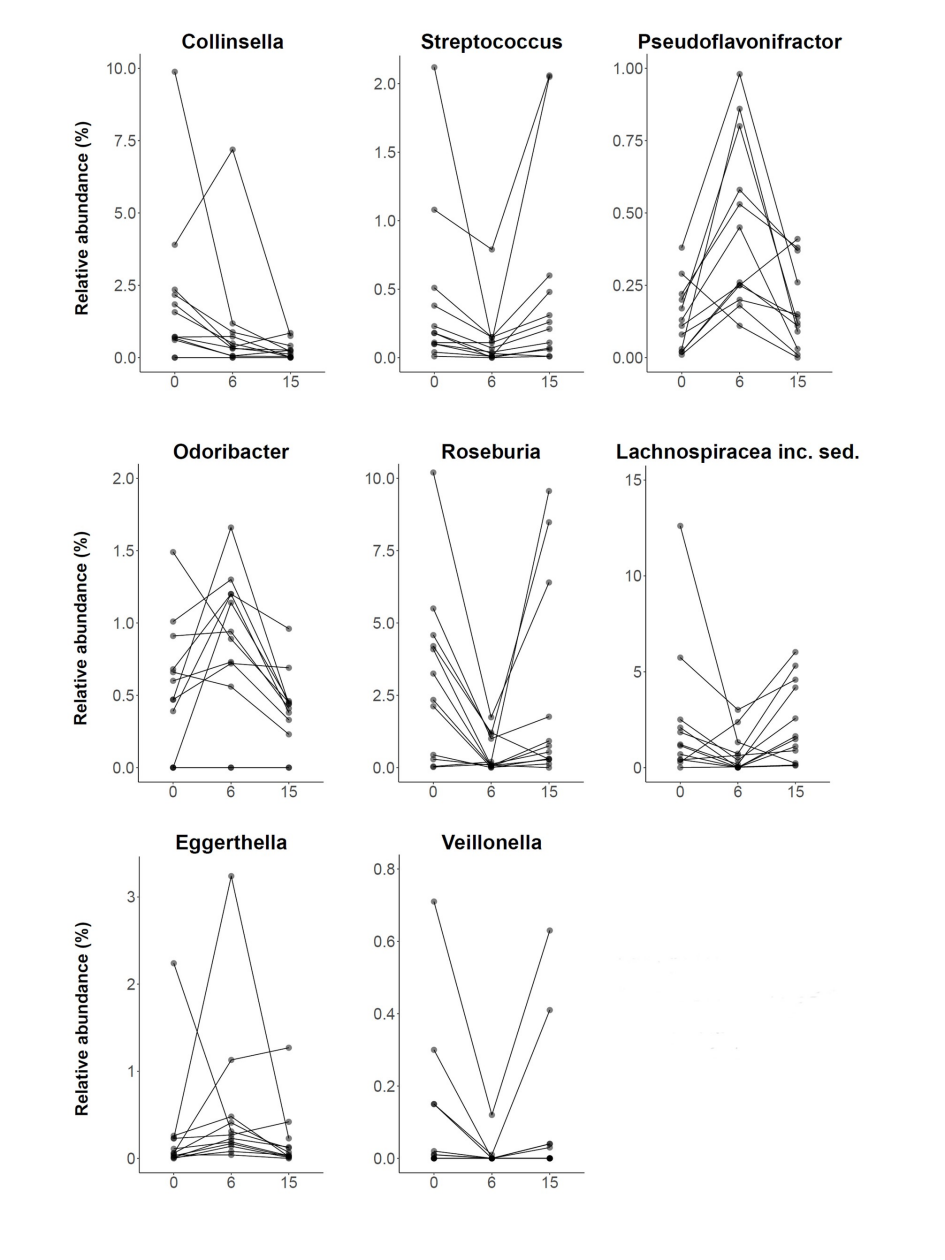
Genera with significantly different abundance between different time points. Plots are portraying the relative abundance changes of the study group (n = 12) including all significantly altered genera at baseline/0 weeks, after six weeks of low-calorie formula diet and after regular food reintroduction at fifteen weeks. Samples corresponding to one individual are connected by a black line. At the end of the study, only abundance changes of *Collinsella* were sustained. inc. sed.: incertae sedis.

To further investigate the specific changes within the fecal microbiota at different study time points, all classified phyla or genera, respectively, that were present in more than 50% of samples at baseline were analyzed (**S1 Table**). The two most common phyla were *Firmicutes* and *Bacteroidetes* accounting for 87.2% of the total abundance at baseline. In contrast to an earlier study [10], no significant alteration in the abundance of *Bacteroidetes* or the *Firmicutes/Bacteroidetes* ratio were detected. At phylum level, only *Actinobacteria* exhibited a significantly decreased abundance fifteen weeks after the study launch (p = 0.002, post-hoc WT) compared to baseline. **Fig 4** presents the average microbiota community composition at genus level for all three time points. *Bacteroides* and *Faecalibacterium* were the two most abundant genera accounting for 32.2% of the total relative abundance at baseline. **Fig 5** displays all genera with globally significantly different abundances between time points, namely *Collinsella* (q = 0.020, FT), *Streptococcus* (q = 0.020, FT), *Pseudoflavonifractor* (q = 0.020, FT), *Odoribacter* (q = 0.031, FT), *Roseburia* (q = 0.040, FT), *Lachnospiraceae incertae sedis* (q = 0.040, FT), *Eggerthella* (q = 0.040, FT), and *Veillonella* (q = 0.040, FT). However, almost all genera which showed a shift in abundance at six weeks compared to baseline (*Streptococcus*, p = 0.004; *Pseudoflavonifractor*, p = 0.003; *Roseburia*, p = 0.002; *Lachnospiraceae incertae sedis*, p = 0.034; *Eggerthella*, p = 0.045; and *Veillonella*, p = 0.022; post-hoc WT), returned to their initial level after fifteen weeks. At the end of the study, only *Collinsella* still exhibited an 8.4-fold lower abundance compared to baseline (p = 0.006, post-hoc WT).

## Discussion

This study investigated the effect of a structured weight loss program on intestinal microbiota composition determined by 16S rRNA gene sequencing in obese type 2 diabetics. To this end, fecal samples of twelve individuals were analyzed at baseline, after six weeks of a low-calorie formula diet and finally after reintroduction of a regular diet fifteen weeks after the start of the program. All selected participants showed continuous weight loss throughout the program accompanied by alterations in the fecal microbiota. These changes were most pronounced after six weeks but partially reversed until the end of the study.

Cross-sectional studies have shown lower microbiota diversity in obese subjects compared to lean controls [8]. This was supported by a reported association of low gut bacterial richness with a higher degree of obesity, insulin resistance and dyslipidemia [9]. In addition, obese individuals with low bacterial richness had experienced greater weight gain in the past compared to obese individuals with high bacterial richness. Decreased microbial diversity has also been identified as a key feature in other medical conditions such as Crohn's disease [26]. Interestingly, fecal microbiota transfer from healthy donors to Crohn's disease patients resulted in an increase of microbial diversity which positively correlated with the reduction of symptoms [27]. Another study investigating the rate of chemotherapy-related blood stream infections in non-Hodgkin lymphoma patients showed an increased rate of blood stream infections in individuals with decreased baseline gut microbiota diversity [28]. The above mentioned investigations suggest low microbial diversity to be disadvantageous for an individual's health and associated with adverse outcomes in disease. Considering the conditions that can arise in obese individuals, it is of interest whether a reduced microbial diversity can be improved by dietary intervention and/or weight loss. To this end, a study reported that microbial gene richness in individuals with previously low genetic bacterial richness increases during a dietary intervention [15]. In our study we can confirm a rise in phylogenetic diversity in parallel with weight loss, which is sustained after the termination of the dietary intervention and reintroduction of a regular diet. However, the increase of the Simpson diversity number after completion of the program was not significant, which might be attributed to the smaller sample size of our group.

Another supposed key feature in the microbiome of patients with obesity is a reduction of the phylum *Bacteroidetes* [8]. This was backed by an observation made under a low-calorie diet, revealing an increasing amount of *Bacteroidetes* in overweight individuals during weight loss [10]. A further study investigated the microbiota alterations after weight loss achieved by diet to that observed after obesity surgery, finding a decrease of *Bacteroidetes* after low-calorie diet in contrast to increasing levels after gastric sleeve resection [29]. Of note, the achieved weight loss was similar in both groups. Another dietary intervention study in obese found neither the amount of *Bacteroidetes* to be different at baseline, compared to lean controls, nor a change in the relative proportion of *Bacteroidetes* after low-calorie diet [11]. *Pataky et al*. likewise found no alteration of *Bacteroidetes* after low-calorie diet induced weight loss in a cohort of obese individuals suffering from fatty liver disease [30]. In the present study, we could also not replicate a significant change of *Bacteroidetes* at any time point during the investigation. The heterogeneity of the results published so far therefore challenge the assumption of reduced *Bacteroidetes* counts being a potentially reversible key feature of obese individuals.

We found eight genera to be significantly altered in their abundance during the study. Seven of them were drastically increased or decreased at six weeks after completion of the low-calorie formula diet. Subsequent reintroduction of a regular diet reversed almost all of the changes until final sample collection at fifteen weeks. This observation confirms two previously published dietary intervention studies which enrolled obese individuals into a very low-calorie diet and showed that large parts of the microbial alterations seen during the dietary intervention regressed back towards baseline after termination of the dietary intervention, irrespective of the weight loss achieved [17, 31]. It is known that dietary modifications themselves directly affect the gut microbial community structure [32]. Hence, our results may suggest that a large proportion of the microbiota changes seen after six weeks of weight loss were mainly driven by the caloric and nutritional content of the low-calorie formula diet rather than being the consequence of the concomitant weight loss which continued over all study time points. It remains unclear whether the observed changes in microbiota composition were a promoting factor for the concomitant weight loss or whether they merely represent a correlating effect of the specific diet. In the present study, the only taxon found to remain consistently associated with weight loss at study termination was the genus *Collinsella* which decreased 8.4-fold. A role of *Collinsella* in a variety of disorders has previously been proposed in other studies. Increased levels of *Collinsella* were found in individuals suffering from type 2 diabetes mellitus and in patients with symptomatic atherosclerotic carotid artery stenosis [33],[34]. Furthermore, in overweight pregnant women the presence of *Collinsella* was associated with increased levels of insulin, triglycerides and very low density lipoproteins (VLDL) [35]. Even in healthy adults elevated *Collinsella* concentrations have been reported to be associated with increased cholesterol and LDL [36]. In infants, an earlier acquisition of a *Collinsella*- and *Bifidobacterium*-dominated microbiome was found to be associated with higher subscapular skinfold thickness, indicating more body fat [37]. In view of the above mentioned studies, the lasting reduction of *Collinsella* during the present weight loss intervention may have contributed to the increased insulin sensitivity at the end of the study. A lower abundance of *Collinsella* may also decrease the susceptibility for cardiovascular disease if it were to be maintained over longer time periods. However, due to the descriptive nature of our study, the reduced abundance of *Collinsella* after weight loss could merely represent a biomarker indicating an improved metabolic state. Nevertheless, these results imply an interesting target genus for future microbiome intervention studies, which would have to determine whether a reduction in *Collinsella* can improve metabolic parameters irrespective of the subject's weight.

Although several studies have addressed changes in gut microbiota during and after weight loss, some of the results have not been consistent and sometimes contradictory. Different results between studies might be explained by the heterogeneous genetic background of the study populations, diverse dietary regimens, different baseline microbial community structure [15] or different (sequencing) methodology [38]. Limitations of the current study include the small sample size and the confined time period of fifteen weeks. It remains unknown whether the reported changes of the microbiota persist in the long term.

To summarize, we confirm earlier reports of increased microbial diversity following weight loss, which in the present study was accompanied by a decrease of *Collinsella*, a genus that has recently been associated with poor metabolic states, type 2 diabetes mellitus, and atherosclerosis. Future intervention studies will have to determine the relevance of *Collinsella* in the pathogenesis of metabolic disorders. This study also shows that dietary interventions carry the potential to directly modify the microbial community composition. Future studies should therefore compare different formulations of hypocaloric diets to characterize their associated microbiota changes and thus their potential for making weight loss programs more effective.

## Supporting information

S1 Table Phylum and genus comparisons. (XLSX)

S2 Table Phenotypic variables. (TXT)

S3 Table Microbiome dataset. (TXT)

S1 Fig Metabolic laboratory parameters. (PDF)

S1 File Study protocol german. (PDF)

S2 File Study protocol english. (PDF)

S3 File TREND checklist. (PDF)

## Abbreviations

BMI: body mass index
FT: Friedman test
HDL: high density lipoprotein
LDL: low density lipoprotein
N2: Simpson diversity number
OTU: operational taxonomic unit
PCoA: principal coordinate analysis
PD: phylogenetic diversity
SHIP: Study of Health in Pomerania
VLDL: very low density lipoprotein
WT: Wilcoxon signed-ranks test.
