# Supplementary material for: A structured weight loss program increases gut microbiota phylogenetic diversity and reduces levels of Collinsella in obese type 2 diabetics: A pilot study
Source: PLoS One. 2019 Jul 18;14(7):e0219489. doi: 10.1371/journal.pone.0219489 (PMC6638920; doi:10.1371/journal.pone.0219489)
Supplement: S1 Fig — (PDF) [file pone.0219489.s004.pdf]

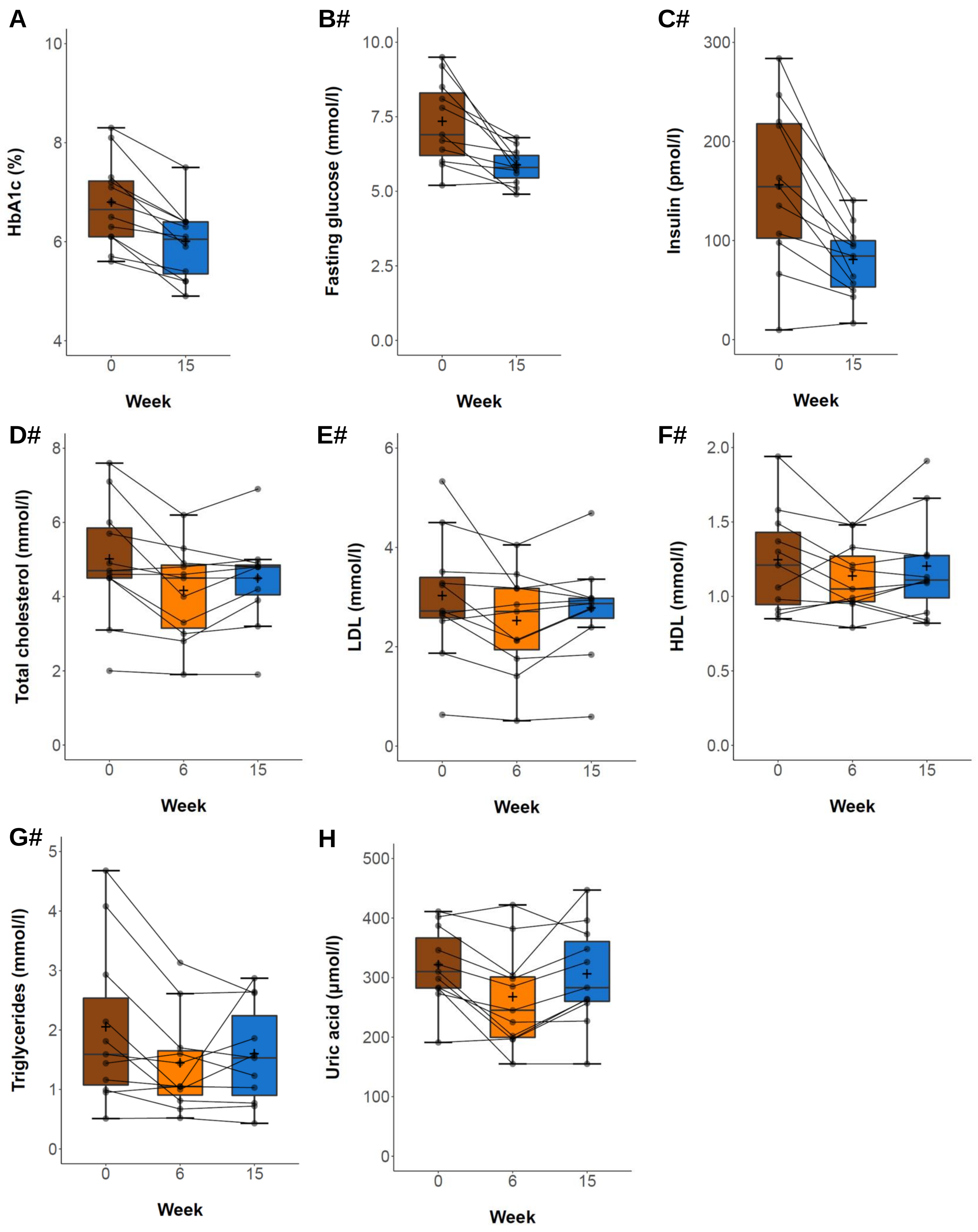

S1 Fig. Development of metabolic laboratory parameters over the study course. Boxplots are portraying the distributions of **(A)** HbA1c, **(B)** fasting glucose, **(C)** insulin, **(D)** total cholesterol, **(E)** low density lipoprotein (LDL), **(F)** high density lipoprotein (HDL), **(G)** Triglycerides, and **(H)** uric acid. At each time point paired samples of 12 participants were evaluated. # one participant was not fasting before the blood sample was obtained and therefore excluded. Corresponding samples at each time point are connected by a black line. + signs indicate the means.
