# Supplementary material for: A structured weight loss program increases gut microbiota phylogenetic diversity and reduces levels of Collinsella in obese type 2 diabetics: A pilot study
Source: PLoS One. 2019 Jul 18;14(7):e0219489. doi: 10.1371/journal.pone.0219489 (PMC6638920; doi:10.1371/journal.pone.0219489)
Supplement: S1 File — (PDF) [file pone.0219489.s005.pdf]

# Studienprotokoll

# Evaluation eines strukturierten Gewichts-reduktionsprogramms zur Therapie von Adipositas und Diabetes mellitus Typ 2

# TADIA-Studie

Universitätsmedizin Greifswald
Klinik für Innere Medizin A
Bereich Ernährungsmedizin
Ferdinand-Sauerbruch-Str.
17475 Greifswald
Tel.: 03834/86-6690 oder -7268

# Inhaltsverzeichnis


**Inhaltsverzeichnis** .......... 2

**Zusammenfassung** .......... 3

**Studienorganisation und Verantwortlichkeiten** .......... 5

**Studienziele** .......... 7

Primärer Zielparameter .......... 7

Sekundäre Zielparameter .......... 7

**Studienteilnehmer** .......... 8

Einschlusskriterien .......... 8

Ausschlusskriterien .......... 8

**Hintergrundinformationen** .......... 9

Adipositas und Diabetes mellitus Typ 2 .......... 9

Das OPTIFAST®-Programm .......... 11

**Methoden** .......... 13

Ermittlung des Ernährungszustandes .......... 13

Messung der klinisch relevanten Parameter .......... 13

Anthropometrie .......... 13

Bioelektrische Impedanzanalyse .......... 13

Erfassung der Ernährungsgewohnheiten .......... 14

HOMA-Index .......... 14

Magnetresonanztomographie-Untersuchung .......... 15

Erfassung der Lebensqualität .......... 15

Erfassung der Schlafqualität .......... 15

**Planung des Studienumfangs und der Auswertung** .......... 16

Studienbeginn .......... 16

Studienablauf .......... 16

Abbruch der Behandlung .......... 17

Studienfinanzierung .......... 18

Statistische Überlegungen .......... 18

**Ethik und Datenschutz** .......... 18

**Literaturverzeichnis** .......... 19

# Zusammenfassung


**Titel: Evaluation eines strukturierten Gewichtsreduktionsprogramms zur Therapie von Adipositas und Diabetes mellitus Typ 2**

| | |
|---|---|
| Intervention: | strukturiertes Gewichtsreduktionsprogramm OPTIFAST® bei adipösen Diabetikern |
| Studiendesign: | bizentrische, retrospektive/prospektive, longitudinale Interventionsstudie |
| Studienpopulation: | Patienten mit BMI 27-45 kg/m² und Diabetes mellitus Typ 2 |
| Patientenzahl: | 35 Patienten |
| Studiendauer: | 15 Wochen (Inventionsphase) |
| Interventionsgruppe: | Adipöse Diabetiker, OPTIFAST® |
| Primärer Zielparameter: | $HbA_{1c}$-Wert |
| Sekundäre Zielparameter: | Stoffwechseleinstellung durch Parameter: Insulinsensitivität, Blutlipidspiegel, Blutdruck, Leberwerte, Schilddrüsenhormone, Albumin, Kreatinin, Harnstoff<br>Fettgehalt und Größe der Leber im Verlauf<br>Fettgehalt der Bauchspeicheldrüse im Verlauf<br>Sekundärerkrankungen des Diabetes mellitus Typ 2<br>Viszerales und subkutanes Fett<br>Medikamentenverbrauch<br>Ernährungsweise<br>Lebensqualität<br>Schlafqualität |

Behandlungssicherheit:

Die während der ersten sechs Wochen eingesetzte sehr niedrig-kalorische (800 kcal), eiweißmodifizierte, vollbilanzierte Formuladiät, orientiert sich in der Zusammensetzung an der EU-Richtlinie und dem aktualisierten § 14 der Diätverordnung. Sie besteht aus 70 g biologisch hochwertigem Eiweiß, 15 g Fett zur Deckung des Bedarfs an mehrfach ungesättigten Fettsäuren und ca. 96 g Kohlenhydraten. Vitamine, Spurenelemente sowie Mineralien sind in ausreichenden und den Vorschriften entsprechenden Mengen zugesetzt. Nach der modifizierten EU Richtlinie der Diätverordnung von 05.05.1999 wird eine ärztliche Kontrolle unterhalb einer Energiezufuhr von 1000 kcal / Tag gefordert und eingehalten.

# Studienorganisation und Verantwortlichkeiten

## Studienleiter

- Prof. Dr. M. Lerch (Klinik für Innere Medizin A, Universitätsmedizin Greifswald)
- OÄ Dr. med. A. Steveling (Klinik für Innere Medizin A, Universitätsmedizin Greifswald)
- M. Sc. oec. troph. L. Vogt (Klinik für Innere Medizin A, Universitätsmedizin Greifswald)

## Prüfärzte

- Dr. med. M. Kraft (Diakoniekrankenhaus Rotenburg an der Wümme)
- Dr. med. J.-P. Kühn (Institut für Diagnostische Radiologie und Neuroradiologie, Universitätsmedizin Greifswald )
- Dr. med. A. Steveling (Klinik für Innere Medizin A, Universitätsmedizin Greifswald)
- Prof. Dr. med. M. Masin (Klinik und Poliklinik für Innere Medizin B, Universitätsklinikum Münster)

## Unabhängige Daten und Sicherheitsbestimmungen

- Dr. rer. nat. E. Weber (Klinik für Innere Medizin A, Universität Greifswald)

## Statistiker

- Dr. Peter Meffert, Institut für Community Medicine / SHIP-KEF

## Kontaktadressen

Prof. Dr. med. Markus M. Lerch
Klinik für Innere Medizin A
Ferdinand-Sauerbruch-Str.
17475 Greifswald
Tel.: 03834/867230
E-Mail: lerch@uni-greifswald.de

Dr. med. Antje Steveling
Klinik für Innere Medizin A
Ferdinand-Sauerbruch-Str.

17475 Greifswald
Tel.: 03834/866675
E-Mail: antje.steveling@uni-greifswald.de

Dr. med. Jens-Peter Kühn
Institut für Diagnostische Radiologie und Neuroradiologie
Ferdinand-Sauerbruch-Str.
17475 Greifswald
Tel.: 03834/862170
E-Mail: kuehn@uni-greifswald.de

Dr. med. Matthias Kraft
Elise-Averdieck-Str. 20
27356 Rotenburg an der Wümme
Tel.: 04261/77-2090
E-Mail: kraftm@diako-online.de

Prof. Dr. Markus Masin
Klinik und Poliklinik für Innere Medizin B
Universitätsklinikum Münster
Albert-Schweitzer-Str. 33
48129 Münster

Dr. Peter Meffert
Universitätsmedizin Greifswald, Institut für Community Medicine / SHIP-KEF
Walther-Rathenau-Str. 48
17475 Greifswald
Tel.: 03834/8619579

M. Sc. oec. troph. Lena J. Vogt
Klinik für Innere Medizin A
Ferdinand-Sauerbruch-Str.
17487 Greifswald
Tel.: 03834/867268
E-Mail: lena.vogt@stud.uni-greifswald.de

## Studienziele

Im Rahmen einer bizentrischen und prospektiven Interventionsstudie an der Klinik für Innere Medizin A der Universitätsmedizin Greifswald und der Klinik für Innere Medizin B des Universitätsklinikums Münster soll der Einfluss einer multimodalen Interventionstherapie, basierend auf einem strukturierten Gewichtsabnahmeprogramm auf die Stoffwechseleinstellung bei adipösen Typ 2 Diabetikern untersucht werden.

### *Primärer Zielparameter*

Es wird davon ausgegangen, dass durch das strukturierte Gewichtsreduktionsprogramm neben dem Gewicht auch der $HbA_{1c}$–Wert sinkt. Dieser stellt mit einer Reduktion von etwa -15% den primären Endpunkt dar.

### *Sekundäre Zielparameter*

- Stoffwechseleinstellungen bei Diabetes mellitus Typ 2:
  - Insulinsensitivität (HOMA-Index)
  - Serum-Plasmaspiegel von Gesamt-, LDL- und HDL-Cholesterin
  - Serum-Plasmaspiegel von TG, Albumin, ALAT, ASAT, GGT, Kreatinin, Harnstoff, C-Peptid, Proinsulin, IGF-1, STH, SHBG Testosteron, SHBG DHEAS, TSH, $T_3$, $T_4$
  - Lebergröße und Fettgehalt der Leber (MRT)
  - Fettgehalt der Bauchspeicheldrüse (MRT)
- Sekundärerkrankungen des Diabetes mellitus Typ 2: Mikroalbuminurie, diabetisches Fußsyndrom, Polyneuropathie, Blutdruck
- Verbrauch oraler Antidiabetika / Insulin
- Ernährungsweise (OptiDiet®)
- Differenzierung viszerales und subkutanes Fettgewebe (MRT/BIA)
- Lebensqualität (SF-12)
- Schlafqualität (Pittsburgh Schlafqualitätsindex)

## Studienteilnehmer

Der Einschluss der Patienten erfolgt nach Überprüfung der Einschlusskriterien durch die Universitätsmedizin Greifswald und dem Universitätsklinikum Münster. Jeder Patient, von dem Material gewonnen wird, erhält einen Identifikationscode und wird in der Zentrale Greifswald registriert. Es werden Blutproben entnommen und Fragebögen vom Patienten ausgefüllt. Außerdem wird für jeden Patienten ein Erfassungsbogen erstellt, in dem der ID-Code eingetragen wird. Der Erfassungsbogen enthält die Einverständniserklärung, Patientenstammdaten, Diagnose, Therapie sowie Angaben über das Blut.

### *Einschlusskriterien*

- BMI > 27 kg/m² und < 45 kg/m²
- Diabetes mellitus Typ 2
- Alter zwischen 18 und 70 Jahren
- Vorliegen der Einverständniserklärung
- Vorliegen der $HbA_{1c}$–Werte und des Gewichts der letzten drei Monaten

### *Ausschlusskriterien*

- Einnahme von Inkretin-Analoga ≤ 3 Monate vor Einschluss
- Hochgradige Nieren- oder Leberinsuffizienz (KDOQI > 3; Child Pugh > A)
- Herzinsuffizienz (NYHA > 3)
- Dokumentierte Essstörung
- Geistige Retardierung, Demenz
- Immobilität
- Alter unter 18 Jahre
- Schwangerschaft / Stillzeit
- Florider Alkohol- und / oder Drogenkonsum
- Ausschluss von der MRT-Untersuchung: Herzschrittmacher, Platzangst, metallhaltige Fremdkörper

Als Kontrolle werden retrospektiv die Veränderungen des Gewichts und der $HbA_{1c}$-Werte der Studienteilnehmer genutzt.

# Hintergrundinformationen

## *Adipositas und Diabetes mellitus Typ 2*

Bei Adipositas handelt es sich um eine pathologische Vermehrung der Körperfettmasse (BMI > 30 kg/m²), die mit einer Gesundheitsgefährdung einhergeht [1]. Laut Nationaler Verzehrsstudie II sind mehr als 68 % der Männer und etwa 50 % der Frauen zwischen 18 und 80 Jahren in Deutschland übergewichtig, davon 21 % bzw. 20 % adipös [2]. Gleichzeitig leben nach Daten des statistischen Bundesamtes und der Study of Health in Pomerania (SHIP-Studie) in Mecklenburg-Vorpommern im Vergleich zu anderen Bundesländern die meisten übergewichtigen Erwachsenen mit einem Anteil von 52,8 % [3][4]. Aufgrund dieser Zahlen sowie einer steigenden Inzidenz und Prävalenz muss Adipositas mit ihren Folgeerkrankungen als eines der größten Gesundheitsprobleme Deutschlands gesehen werden. Es besteht ein deutlich erhöhtes Morbiditäts- und Mortalitätsrisiko, woraus die Indikation zu einer lebenslangen Betreuung der Patienten resultiert [5].

Große prospektive Studien haben belegt, dass ein steigender BMI mit einer Vielzahl von Begleit- und Folgeerkrankungen sowie mit einer zunehmenden Verkürzung der Lebenserwartung verbunden ist [1][6]. Die durchschnittliche Lebenserwartung bei Vorliegen von Adipositas im Alter von 40 Jahren, ohne weitere Risikofaktoren wie Rauchen, ist um sieben Jahre reduziert [7]. Nicht jeder Übergewichtige entwickelt zwangsläufig Folgeerkrankungen, allerdings steigt das Risiko mit dem Ausmaß und der Dauer der Adipositas [1]. Die wichtigsten Komorbiditäten und Komplikationen bei Adipositas sind arterielle Hypertonie, kardiovaskuläre Erkrankungen, Diabetes mellitus Typ 2, Krebserkrankungen und die Entwicklung einer Fettleber [8].

Menschen mit Adipositas haben ein bis zu 5-fach erhöhtes Risiko einen Diabetes mellitus zu entwickeln [9][10]. Der Terminus Diabetes mellitus umfasst eine heterogene Gruppe von Erkrankungen des Glukosestoffwechsels, die durch eine pathologische Kontrolle des Zuckerstoffwechsels mit konsekutiver Blutzuckererhöhung charakterisiert werden. Hierbei ursächlich ist ein Fehlen von Insulin oder eine ungenügende Insulinwirkung (Insulinresistenz). 80 % aller Diabetiker weisen einen Typ 2 Diabetes auf. Hierbei ursächlich ist eine vorwiegende Insulinresistenz mit relativem Insulinmangel bis hin zu einem vorwiegenden sekretorischen Defekt mit Insulinresistenz [11].

Insulin ist ein Peptidhormon und wird in den β-Zellen der Langerhans'schen Inseln des Pankreas sezerniert. Die durch Exocytose der β-Granula ausgelöste Insulinsekretion ist

von der Höhe der Glukosekonzentration im extrazellulären Raum abhängig. Steigt diese über den im Nüchternzustand vorliegenden Wert von etwa 4 mmol/l an, kommt es rasch zu einer Insulinfreisetzung. Bis zu einer Glukosekonzentration von ungefähr 15 mmol/l ist die Menge freigesetzten Insulins proportional zur Glukosekonzentration. Neben der Glukose lösen außerdem verzweigtkettige Aminosäuren, gastrointestinale Hormone sowie Pharmaka, beispielsweise die in der Diabetes-Therapie verwendeten Sulfonylharnstoffe, eine Insulinsekretion aus. Dagegen bilden Catecholamine wichtige physiologische Hemmstoffe der Insulinsekretion. Die Insulinwirkung wird durch die Bindung an den Insulinrezeptor ausgelöst, wodurch die Glukoseaufnahme durch Translokation des Glukosetransporters GLUT-4 in die Plasmamembran der Skelettmuskulatur und der Leber stimuliert wird. Nach der Resorption wird die Glukose für die Synthese von Glykogen und damit zur Energiespeicherung verwendet. Gleichzeitig kommt es durch die gesteigerte Glukoseaufnahme zu einem Absinken der Glukosekonzentration im Blut [12].

Bei adipösen Menschen tritt häufig eine Glukoseintoleranz auf, bei der die Glukoseverwertung im Muskel reduziert ist (periphere Insulinresistenz) und in der Leber eine vermehrte Glukoneogenese stattfindet (hepatische Insulinresistenz). Im Frühstadium erfolgt die Kompensation durch eine vermehrte Freisetzung von Insulin mit begleitender Hyperinsulinämie. Im Laufe der Zeit besteht die Gefahr einer Abnahme der sekretorischen Funktion der β-Zellen des Pankreas [13]. Bei ungenügender Einstellung des Diabetes kann es zu Spätkomplikationen kommen, zu denen kardiovaskuläre Erkrankungen, Retinopathie, diabetische Nephropathie bis hin zur dialysepflichtigen Niereninsuffizienz, diabetische Polyneuropathie, Amputationen sowie zu Störungen des autonomen Nervensystems mit Paresen im Gastrointestinaltrakt zählen [14].

Fachgesellschaften empfehlen bei Adipositas zunächst Lebensstil-Interventionen, bevor medikamentöse oder operative Therapien in Frage kommen [8][9]. Programme mit einer umfassenden Betreuung durch Ärzte, Verhaltenstherapeuten, Ernährungsfachkräften und Sporttherapeuten, häufig in Kombination mit einer Formula-Nahrung im Rahmen eines strukturierten Gewichtsabnahmeprogrammes zeigen gute Ergebnisse bezüglich einer nachhaltigen Gewichtsreduktion [15][16][17]. Solche intensiv betreute Lebensstil-Interventionen können auch bei Diabetikern erfolgreich sein und die Diabetes-assoziierte Mortalität senken [15][18]. Allerdings beschäftigen sich bislang viele Wissenschaftler eher mit Lebensstil-Interventionen als Prävention von Diabetes mellitus Typ 2 und nicht als Therapie [19][20][21]. Aus diesen Gründen wird an der Klinik und Poliklinik für Innere Medizin A der Universitätsmedizin Greifswald und der Klinik für Innere Medizin B des Universitätsklinikums Münster das OPTIFAST®II-Kurzprogramm mit Typ 2 Diabetikern

durchgeführt. So erhalten übergewichtige Diabetiker die Chance, Gewicht zu verlieren und eine Verbesserung der Stoffwechseleinstellung zu erreichen. Durch zusätzliche Erhebungen werden die Insulinsensitivität sowie der Fettgehalt der Leber und der Bauchspeicheldrüse im Verlauf der Gewichtsreduktion beobachtet.

## *Das OPTIFAST®-Programm*

In einer einwöchigen Vorbereitungsphase wird eine ausgiebige Gesundheitsanalyse durchgeführt, um die Einschlusskriterien zu überprüfen und eventuelle Gesundheitsrisiken auszuschließen. Diese beinhaltet Laboranalysen, Erstellung eines kardiovaskulären Risikoprofils sowie die Detektion von Erkrankungen des metabolischen Syndroms, zu denen Stoffwechselstörungen, gestörte Glukosetoleranz, Fettleber und hormonelle Störungen gehören. In dieser Phase erfolgt die Analyse des bisherigen Ernährungs- und Bewegungsverhaltens mit Hilfe der in der ernährungsmedizinischen Ambulanz etablierten Methoden (Erläuterungen im Kapitel Methoden).

An diese Vorbereitungsphase schließt sich eine Fastenphase von sechs Wochen an. Gleichzeitig wird mit dem Patienten ein individuelles Bewegungsprogramm erarbeitet. Die Kost während der Fastenphase besteht aus einer sehr niedrig-kalorischen (800 kcal), eiweißmodifzierten, vollbilanzierten Formuladiät, um eine größtmögliche Fettreduzierung unter weitestgehender Beibehaltung der fettfreien Körpermasse zu erreichen. Diese orientiert sich in der Zusammensetzung an der EU-Richtlinie und dem aktualisierten § 14 der Diätverordnung. Sie besteht aus 70 g biologisch hochwertigem Eiweiß, 15 g Fett zur Deckung des Bedarfs an mehrfach ungesättigten Fettsäuren und ca. 96 g Kohlenhydraten. Vitamine, Spurenelemente sowie Mineralien sind in ausreichenden und den Vorschriften entsprechenden Mengen zugesetzt. Nach der modifizierten EU-Richtlinien der Diätverordnung vom 05.05.1999 wird eine ärztliche Kontrolle unterhalb einer Energiezufuhr von 1000 kcal / Tag gefordert und eingehalten.

In der sich daran anschließenden 4-wöchigen Umstellungsphase erfolgt die schrittweise Umstellung auf eine energiereduzierte ausgewogene Mischkost nach den Leitlinien der „Deutschen Gesellschaft für Ernährung (DGE)“ mit einem Fettanteil von < 30 % und einem Kohlenhydratanteil von > 50-55 %. Es werden individuelle Ernährungspläne erstellt, mit denen sich der Patient identifizieren und wohlfühlen kann. Gleichzeitig wird das Bewegungsprogramm ausgebaut und vertieft.

Mit der 11. Woche beginnt die 5-wöchige Stabilisierungs- und Intensivierungsphase, in der die Teilnehmer das neu erlernte Ess- und Bewegungsverhalten nachhaltig trainieren und vertiefen. Diese Phase stellt den zentralen Kernpunkt des Programms dar, da erfahrungs-

gemäß in dieser Zeit der Rückfall auf rigide Verhaltensmuster am häufigsten ist. Hier kommt der Ernährungsberatung sowie dem psychologischen Konzept der flexiblen Verhaltenskontrolle mit einem Pyramiden-Training eine besondere Bedeutung zu.

Im Verlauf des 15-wöchigen Programms finden einmal wöchentlich Gruppenschulungen (12 Teilnehmer) von einer durchschnittlichen Dauer von 90 Minuten statt. Diese werden von geschulten Ernährungsmedizinern, Ernährungswissenschaftlern, Diätassistenten, Psychologen und Physiotherapeuten gehalten. Das OPTIFAST® Programm legt in einem detaillierten, konkreten Therapie-Manual die Inhalte für jede Woche fest. Die Kernthemen umfassen die Verhaltenstherapie, Ernährungsberatung, Bewegung, Kochkurse sowie die Schulung hinsichtlich medizinischer Themenschwerpunkte. Es enthält zudem Arbeitsblätter, Zusammenfassungen und spezielle Videos, die grundlegende Informationen verständlich darstellen. Durch diese Medien, die auch jeder Patient erhält, wird eine hohe Standardisierung der Inhalte und Aussagen gewährleistet. Durch die schriftlichen Unterlagen und Kommunikation mit dem Gruppentherapeuten in den Sitzungen werden schrittweise Trainingsaufgaben vereinbart, die wöchentlich fortschreiten. Diese Therapieelemente sind lernpsychologisch fundiert und erfüllen die Anforderungen einer verhaltenstherapeutischen Intervention.

Ziel ist eine langfristige Gewichtsstabilisierung durch Erlernen und Trainieren eines gesunden und aktiven Lebensstils. Die Konzeption des Therapiemodells erfüllt alle Ansprüche, die nach neuesten Erkenntnissen an die Therapie der Adipositas zu stellen sind: Training in flexibler Verhaltenskontrolle, Abbau rigider Verhaltensstrategien, Steigerung der aktiven Bewegung im Alltag, realistische Anspruchsniveausetzung als Misserfolgsprophylaxe, soziale Unterstützung durch gruppendynamische Prozesse, permanente ärztliche und psychologische Überwachung sowie langfristige Festlegung des Trainingsprogramms über 15 Wochen mit Anschlussprogramm.

Laboranalysen werden routinemäßig zu vorgegebenen Zeitpunkten während des Programms in den Wochen 0, 3, 7 und 13 durchgeführt und dokumentiert. Außerdem werden sämtliche Adipositas relevanten Untersuchungsergebnisse, insbesondere der Gewichtsverlauf, regelmäßig dokumentiert, um so den Therapieerfolg oder Misserfolg zu überwachen.

# Methoden

## *Ermittlung des Ernährungszustandes*

### Messung der klinisch relevanten Parameter

Die Bestimmung von Glukose, Insulin, $HbA_{1c}$, Triglyceride, HDL-Cholesterin, LDL-Cholesterin, Gesamt-Cholesterin, ALAT, ASAT, GGT, AP, Kreatinin, Harnstoff, Bilirubin, TSH, $T_3$, $T_4$, kleines Blutbild, Natrium, Kalium, Calcium, 25- und 1,25-Vitamin D, Phosphat, Harnsäure, SHBG, Testosteron, IGF-1, LH, Prolaktin gehört zu den Standard-Laboruntersuchungen.

Die Blutröhrchen zur Bestimmung von DNA- und RNA-Expression sowie die Stuhlproben zur DNA-Analyse werden durch das Forschungslabor der Klinik für Innere Medizin A der Universitätsmedizin analysiert und gelagert.

### Anthropometrie

Die Probanden werden während des Gewichtsreduktionsprogramms wöchentlich gewogen, so dass der Body Mass Index aus dem Verhältnis von Körpergewicht zu Körpergröße zum Quadrat [kg/m$^2$] berechnet werden kann. Weiter werden mit einem Maßband der Hüftumfang sowie der Taillenumfang ermittelt. Letzterer wird mittig zwischen dem unteren Rippenbogen und Beckenkamm in normaler Ausatmung gemessen.

### Bioelektrische Impedanzanalyse

Für die Erfassung der Körperzusammensetzung wird bei den Teilnehmern in den Wochen 1, 7 und 15 eine Bioelektrische Impedanzanalyse (BIA) durchgeführt. Diese Analyse ist eine weit verbreitete und nicht-invasive Methode zur Analyse der Körperzusammensetzung und des Ernährungszustandes, welche auf den unterschiedlichen elektrischen Leitfähigkeiten der verschiedenen Gewebetypen beruht. Über je zwei Elektroden an Hand und Fuß wird mittels eines Impedanzanalyse-Gerätes ein schwaches, nicht spürbares Stromfeld im Körper erzeugt. Dabei werden zwei unterschiedliche elektrische Widerstände gemessen, der Wasserwiderstand (R), aus dem das Körperwasser, die Magermasse (fettfreie Masse) und das Körperfett bestimmt werden und der Zellwiderstand (Xc), womit die Muskel- und Organmasse des Körpers angezeigt

wird. Aus diesen Messwerten liefert die zugehörige Software (Data Input®) die komplette Analyse der Körperzusammensetzung. So können mittels der BIA-Körperanalyse die gesamten Bestandteile des Körpers bestimmt und exakt der Anteil von Fett, Muskelmasse und Wasser gemessen werden [22].

## Erfassung der Ernährungsgewohnheiten

Um die Ernährungsgewohnheiten der Teilnehmer vor und nach der Intervention miteinander vergleichen zu können, führen diese in Woche 0 und in Woche 16 ein Ernährungsprotokoll, das mit der Software OptiDiet® ausgewertet wird. OptiDiet® ist ein Programm mit Fachinformationen und Empfehlungen zu einer Vielzahl von Diäten und Allergien. Die Datenbasis bildet der komplette aktuelle Bundeslebensmittelschlüssel, der durch eine Fülle von diätetischen Lebensmitteln ergänzt ist. Die Nährwertangaben spielen insbesondere bei der Berechnung von Ernährungsprotokollen, Plänen und Rezepten eine große Rolle und ermöglichen eine präzise Nährwertberechnung.

Die Nährstoffempfehlungen für Gesunde richten sich im Programm nach den offiziellen DGE-Empfehlungen und sind als Bewertungskurven abgebildet. Bei den einzelnen Diäten werden diese Bewertungskurven entsprechend den diätetischen Zielen und Vorgaben modifiziert. Datengrundlage für die Diäten bilden die verschiedenen Versionen des Rationalisierungsschemas der Deutschen Gesellschaft für Ernährungsmedizin und zahlreiche Schriften und Bücher über Ernährungsmedizin und Diätetik.

## *HOMA-Index*

Zur Berechnung der Insulinsensitivität wird der Homeostasis-Model-Assessment-Insulin-Resistance-($HOMA_{IR}$)-Index verwendet. Dafür werden die Nüchtern-Insulin und Nüchtern-Glukosewerte aus den Blutabnahmen genutzt. Der $HOMA_{IR}$-Index spiegelt die Glukose-Insulin-Homöostase wieder. Der Grenzwert zur Bestimmung einer Insulinresistenz mittels $HOMA_{IR}$-Index liegt für Erwachsene bei > 2,5. Die Berechnung beruht auf der folgenden, vereinfachten Näherungsformel [23]:

$$HOMA_{IR}\text{-Index} = \frac{\text{Nüchtern-Insulin } [\mu U/ml] \times \text{Nüchtern-Glukose } [mmol/l]}{22{,}5}$$

### *Magnetresonanztomographie-Untersuchung*

Für die regelmäßige Quantifizierung von Fett (Leberfett, viszerales Fett, Fettgehalt des Pankreas) ist die Magnetresonanztomographie (MRT 1.5 Tesla Magnetom Avanto, Siemens HealthCare, Erlangen) ein geeignetes Verfahren. Die Messung besteht aus einem standardisierten Sequenzprotokoll, welches einschließlich Lagerung, Einstellung und Kalibrierungsvorgängen ungefähr zehn Minuten dauert. Die MRT ist ein diagnostisches Verfahren, das im Gegensatz zur Computertomographie keine ionisierende Strahlung (Röntgenstrahlung) für die Bilderstellung verwendet, sondern mit Radiowellen und Magnetfeldern arbeitet. Eine Kontrastmittelgabe ist nicht erforderlich. Allgemeine Kontraindikationen werden im Vorfeld der Studie durch einen gesonderten (klinisch akzeptierten) MRT Aufklärungsbogen ausgeschlossen.

### *Erfassung der Lebensqualität*

Für die Erfassung der gesundheitsbezogenen Lebensqualität wird der SF-12 herangezogen. Dabei handelt es sich um eine Kurzform des krankheitsübergreifenden Messinstrumentes SF-36 Health Survey. Es wird sowohl der allgemeine Gesundheitszustand als auch die Beeinträchtigung im Alltag erfasst. Das Ergebnis wird als Skalenwert zwischen 0 und 100 angegeben, wobei eine niedrige Zahl ein schlechtes Befinden bedeutet.

### *Erfassung der Schlafqualität*

Die Schlafqualität vor und nach der Intervention wird mit dem Pittsburgh Schlafqualitäts-Index (PSQI) ermittelt. Dieser erfragt retrospektiv für einen Zeitraum von vier Wochen die Häufigkeit schlafstörender Ereignisse, die Einschätzung der Schlafqualität, die gewöhnlichen Schlafzeiten, Einschlaflatenz und Schlafdauer, die Einnahme von Schlafmedikationen sowie die Tagesmüdigkeit. Der Test gliedert sich in sieben Komponenten, die jeweils einen Wert zwischen 0 und 3 annehmen. Diese Werte werden zu einem Gesamtwert aufaddiert, wobei höhere Werte einer verminderten Schlafqualität entsprechen.

Die diagnostische Validität des Fragebogens ist bezüglich seiner Sensitivität und Spezifität in bisher vier Studien untersucht worden. Die Sensitivität lag immer über 80% (80-100%), die Spezifität, die aber nur in drei Studien untersucht wurde, zeigt ähnlich hohe Werte (83-87%) [24].

# Planung des Studienumfangs und der Auswertung

## *Studienbeginn*

Die Patientenrekrutierung beginnt nach Erhalt eines positiven Ethikvotums durch die Ethikkommission der Universitätsmedizin Greifswald sowie der Universitätsmedizin Münster. Die Studie wird in der NTTC Datenbank angemeldet.

## *Studienablauf*

In Abbildung 1 ist der Studienablauf dargestellt. Vor dem Beginn des Gewichtsreduktionsprogramms OPTIFAST® finden die Patientenaufklärung und die Eingangsuntersuchungen statt. Bevor das Programm in Woche 1 startet, werden eine MRT- und eine BIA-Messung sowie die Labordiagnostik und die anthropometrischen Messungen durchgeführt. Zusätzlich führen die Teilnehmer ein 7-Tage-Ernährungsprotokoll und beantworten einen Fragebogen zur Lebens- und Schlafqualität. Die Erhebungen vor dem Gewichtsreduktionsprogramm werden nach dem Programm in Woche 15 wiederholt.
Das Gewichtsreduktionsprogramm läuft insgesamt 15 Wochen und setzt sich aus dem OPTIFAST®-Kurzprogramm und zusätzlichen Erhebungen zusammen. Wöchentlich wird das Gewicht sowie der Taillen- und Hüftumfang gemessen. Eine BIA- und MRT-Messung findet zusätzlich in Woche 7 statt sowie eine Labordiagnostik in Woche 3 und 7 statt. Die ärztliche Betreuung ist während der gesamten Studie sichergestellt.
Für die Erfassung des langfristigen Erfolges sind Follow-up-Untersuchungen nach 3, 6 und 9 Monaten geplant. Für den Fall, dass die Teilnehmer langfristig ihr neu erreichtes Gewicht nicht halten sollten, ist eine Anschlussstudie mit einer Mahlzeitenersatztherapie vorgesehen.

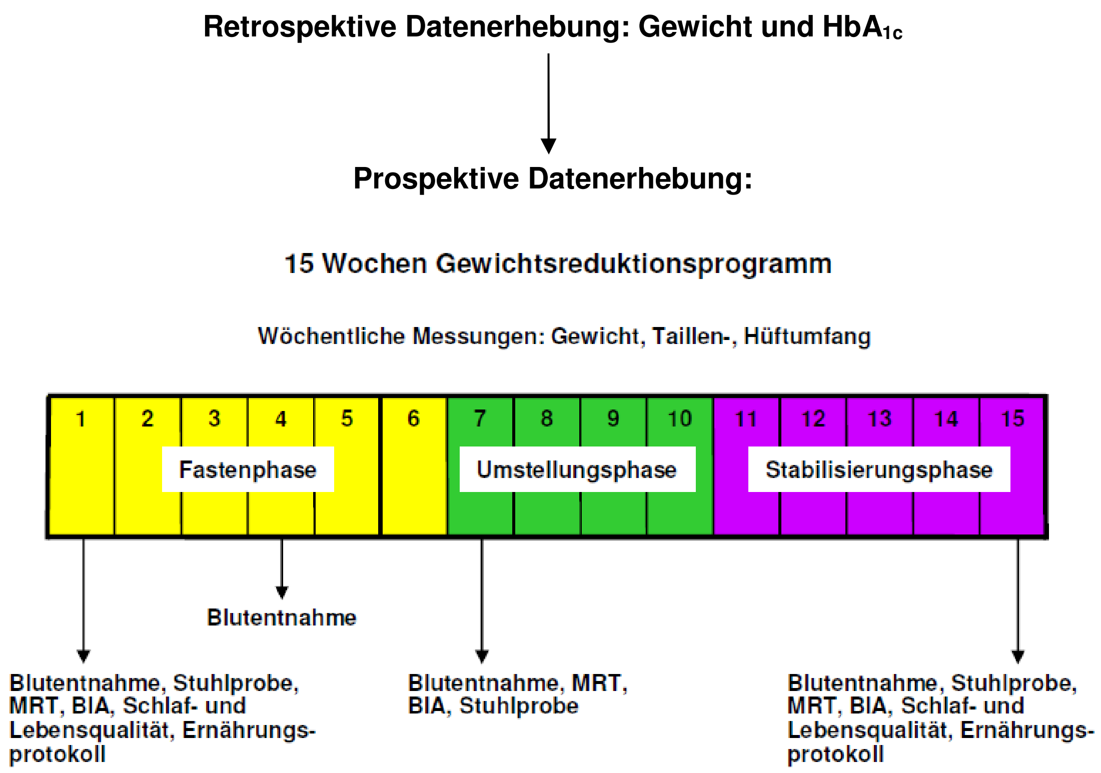


Abbildung 1: Zeitpunkte der Datenerhebung

## *Abbruch der Behandlung*

Die Studie kann unter folgenden Umständen abgebrochen werden:

- Auf Wunsch des Patienten – der Patient kann zu jedem Zeitpunkt frei entscheiden aufzuhören
- Wenn ein Studienleiter der Meinung ist, es wäre nachteilig für den Patienten die Behandlung fortzuführen

Wenn es bei einem Patient zum Abbruch kommen sollte, muss der Studienleiter die genauen Gründe dafür dokumentieren. Wenn möglich sollte die Schlussbeurteilung immer komplettiert werden. Allerdings hat der Patient das Recht sowohl von der gesamten Studie als auch von bestimmten Teilen zurückzutreten. Wenn der Abbruch aufgrund eines bestimmten Ereignisses stattfindet, sollte dieses sorgfältig dokumentiert und der Patient überwacht werden, bis sich die Situation geklärt hat. Im Falle eines Abbruchs wäre es dennoch gut, wenn die Patienten an der Nachuntersuchung teilnehmen, natürlich nur mit deren Einverständnis.

### *Studienfinanzierung*

Die Firma Nestlé unterstützt das Studienvorhaben mit einem Rabatt von -15% auf den Einkaufspreis der OPTIFAST®-Nahrung.

### *Statistische Überlegungen*

Die retrospektiv ermittelten $HbA_{1c}$-Werte vor der Intervention dienen als Kontrollen innerhalb der gepaarten Stichproben. Die Veränderungen vor der Intervention werden mit den Veränderungen durch die Intervention verglichen. Hierzu wird der Wilcoxon-Vorzeichen-Rang-Test angewendet. Sollten die Residuen normalverteilt sein, kann auch ein t-Test für gepaarte Stichproben Verwendung finden. Eine Poweranalyse unter Verwendung von Literaturwerten ergab eine nötige Patientenzahl von n = 35 bei einer Teststärke von 0,8 und n = 57 bei einer Teststärke von 0,95 (Abbildung 2). Die Daten werden mit Hilfe der Software STATA 11 ausgewertet.

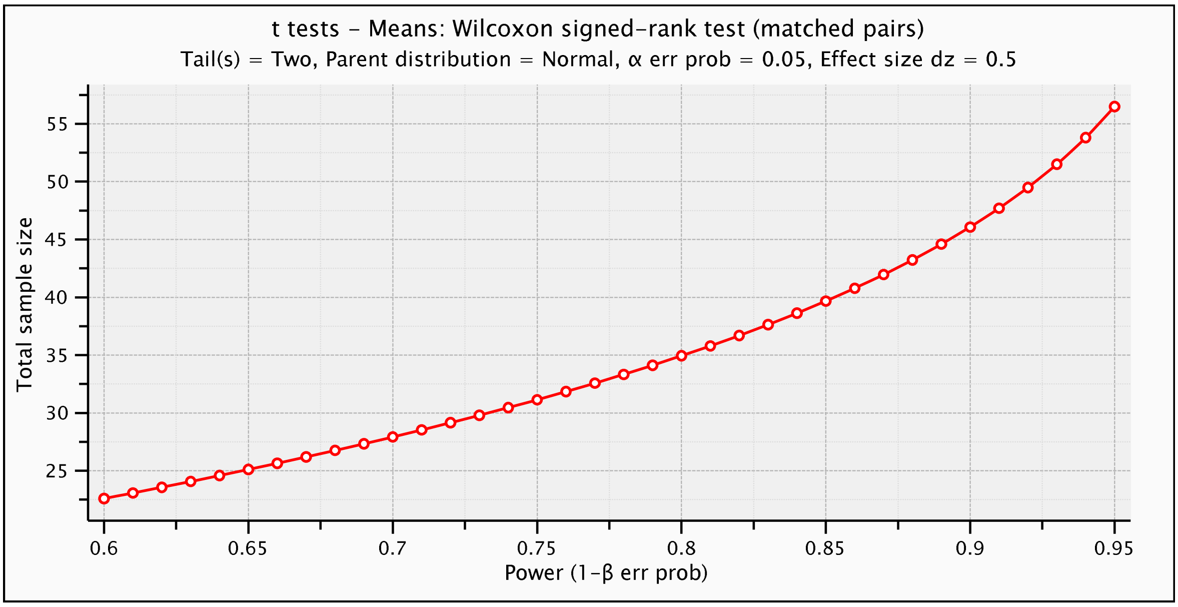


Abbildung 2: Abhängigkeit der benötigten Stichprobengröße von der Teststärke

## Ethik und Datenschutz

Die elektronische Speicherung der Daten erfolgt pseudonymisiert unter einem Identifikationscode. Alle Daten der Materialsammlung werden kennwortgeschützt auf einem Computer der Inneren Medizin A der Uniklinik Greifswald zentral gespeichert. Zugriff auf nicht anonymisierte Daten haben nur die an der Studie beteiligten Mitarbeiter.

# Literaturverzeichnis


1. Hauner H: Adipositas – Klinik und Ernährungstherapie: Schauder P und Ollenschläger G: Ernährungsmedizin – Prävention und Therapie, Urban und Fischer, 2. Auflage: 537-567

2. Bundesministerium für Ernährung, Landwirtschaft und Verbraucherschutz und Max Rubner Institut, Bundesforschungsinstitut für Ernährung und Lebensmittel, Standort Karlsruhe: Nationale Verzehrsstudie II (2005-2009), Stand: 25.05.2011

3. Puls R, Völzke H: Ganzkörper-MRT in der „Study of Health in Pomerania“. Radiologe, 2011; 51:379-383

4. Statistisches Bundesamt, Wiebaden: Körpermaße der Bevölkerung. Mikrozensus – Fragen zur Gesundheit, 2009

5. Aucott L, Gray D, Rothnie H, Thapa M, Waweru C: Effects of lifestyle interventions and long-term weight loss on lipid outcomes – a systematic review. Obesity, 2011; e412-e425

6. Fontaine KR, Redden DT, Wang C, Westfall AO, Allison DB: Years of life lost due to obesity. JAMA, 2003; 289:187-193

7. Haslam DW, James PT: Obesity. Lancet, 2005; 366:1197-209

8. Hauner H, Buchholz G, Hamann A, Husemann B, Koletzko B, Liebermeister H, Wabitsch M, Westenhöfer J, Wirth A, Wolfram G: Prävention und Therapie der Adipositas – Evidenzbasierte Leitlinie. 2007; 1-29

9. Alberti KGMM, Eckel RH, Grundy SM, Zimmet PZ, Cleeman JI, Donato KA, Fruchart JC, James WPTJ, Loria CM, Smith SC: Harmonizing the Metabolic Syndrome: A joint Interim Statement of the International Diabetes Federation Task Force on Epidemiology and Prevention; National Heart, Lung and Blood Institut; American Heart Association and International Association for the Study of Obesity. Circulation, 2009; 120:1640-1645

10. Mack F, Abeygunawardhana N, Mundt T, Schwahn C, Proff P, Spassov A, Kocher T, Biffar R: The factors associated with body mass index in adults from the study of health in Pomerania (SHIP-0), Germany. Journal of Physiology and Pharmacology, 2008; 59:5-16

11. Toeller M: Diabetes mellitus: Schauder P und Ollenschläger G: Ernährungsmedizin – Prävention und Therapie, Urban und Fischer, 2003, 2. Auflage: 713-722

12. Löffler G: Regulation des Intermediärstoffwechsels. Löffler G: Basiswissen Biochemie mit Pathobiochemie, Springer Medizin Verlag, 2005; 6. Auflage:350-356

13. Burkhard M: Adipositas und metabolisches Syndrom. Koula-Jenik H, Kraft M, Miko M, Schulz RJ: Leitfaden Ernährungsmedizin, Urban und Fischer, 2006, 1. Auflage:419-436

14. Gellner R, Hengst K. Diabetes mellitus und Folgeerkrankungen. Koula-Jenik H, Kraft M, Miko M, Schulz RJ: Leitfaden Ernährungsmedizin, Urban und Fischer, 2006, 1. Auflage:455-468

15. Bischoff SC, Damms-Machado A, Betz C, Herpertz S, Legenbauer T, Löw T, JG Wechsler, Bischoff G, Austel A, Ellrott T: Multicenter evaluation of an interdisciplinary 52-week weight loss program for obesity with regard to body weight, comorbidities and quality of life - a prospective study. International Journal of Obesity, 2011

16. Wadden TA, Frey DL: A Multicenter Evaluation of a Proprietary Weight Loss Programm for the Treatmend of Marked Obesity: a five-year follow-up. Int J Eat Disord, 1997; 22:203-212

17. Norris SL, Zhang X, Avenell A, Gregg E, Brown T, Schmid CH, Lau J: Long-term non pharmacological weight loss interventions for adults with type 2 diabetes mellitus . 2005

18. Williamson DF, Thompson TJ, Thun M, Flanders D, Pamuk E, Byers T: Intentional Weight Loss and Mortality among Overweight Individuals with Diabetes. Diabetes Care, 2000; 23:1499-1504

19. Reduction in the incidence of type 2 diabetes with lifestyle intervention or metformin. N Engl J Med, 2002; 346:393-403

20. :Lindström J, Louheranta A, Mannelin M, Rastas M, Salminen V, Eriksson J, Uusitupa M, Toumilehto J. The finnish diabetes prevention study (DPS). Diabetes Care, 2003; 26:3230-3236

21. Lindström J, Eriksson JG, Valle TT, Aunola S, Cepaitis Z, Hakumäki M, Hämäläinen H, Ilanne-Parikka P, Keinännen-Kiukaanniemi S, Laakso M, Louheranta A, Mannelin M, Martikkala V, Moltchanov V, Rastas M, Salminen V, Sundvall, Uusitupa M, Tuomilehto J: Prevention of diabetes mellitus in subjects with impaired glucose tolerance in the finnish diabetes prevention study: results from a randomized clinical trial. J Am Soc Nephrol, 2003; 14:S108-S113

22. Kushner RF, Kunigk A, Alspaugh M, Andronis PT, Leitch CA, Schoeller DA: Validation of bioelectrical-impedance analysis as a measurement of change in body composition in obesity. Am J Clin Nutr, 1990; 52:219-23

23. Matthews DR, Hosker JP, Rudenski AS, Naylor BA, Treacher DF, Turner RC: Homeostasis model assessment: insulin resistance and beta-cell function from fasting plasma glucose and insulin concentrations in man. Diabetologia, 1985; 28:412-419

24. Buysse DJ, Reynolds III CF, Monk TH, Berman SR, Kupfer DJ. The Pittsburgh Sleep Quality Index: A new instrument for psychiatric practice and research. Psychiatry Research 1989, 28:193-213
