# Supplementary material for: A structured weight loss program increases gut microbiota phylogenetic diversity and reduces levels of Collinsella in obese type 2 diabetics: A pilot study
Source: PLoS One. 2019 Jul 18;14(7):e0219489. doi: 10.1371/journal.pone.0219489 (PMC6638920; doi:10.1371/journal.pone.0219489)
Supplement: S2 File — (PDF) [file pone.0219489.s006.pdf]

Study protocol

Evaluation of a standardized Weight-loss Program for Therapy of Obesity and Diabetes mellitus Type 2

TADIA-Study

University Medicine Greifswald
Department of Medicine A
Nutritional Medicine
Ferdinand-Sauerbruch-Str.
17475 Greifswald
Germany
Tel.: +49 3834/86-6690

Table of Contents

Table of Contents **2**
Summary **3**
Study group and responsibilities **5**
Aims **7**
Primary Endpoint 7
Secondary Endpoint 7
Recruitment of the study population **8**
Inclusion criteria 8
Exclusion criteria 8
Background **9**
Obesity and Diabetes mellitus Type 2 9
The OPTIFAST®-Program 10
Methods **13**
Nutritional status 13
Measurement of clinical relevant parameters 13
Anthropometric data 13
Bioelectric impedance analysis 13
OptiDiet 14
HOMA-Index 14
Magnetic resonance imaging 14
Quality of Life 14
Sleep quality 15
Study design **16**
Initiation of the study 16
Study procedure 16
Study termination 17
Financial support 17
Statistical considerations 18
Ethical and data safety issues **18**
References **19**

Summary

Title: Evaluation of a standardized weight-loss program for therapy of obesity and diabetes mellitus type 2

Intervention:	standardized weight-loss program OPTIFAST® for obese diabetes patients
Study design:	dual center retrospective / prospective longitudinal intervention study
Study population:	patients with BMI 27-45 kg/m² and diabetes mellitus type 2
Number of patients:	35 patients
Study period :	15 weeks
Intervention group:	Obese diabetics, OPTIFAST®
Primary endpoint:	HbA_{1c}-value
Secondary endpoints:	Parameters of metabolism: insulin sensitivity, blood lipids, blood pressure, transaminases, thyroid hormone, albumin, creatinine, and urea<br>Liver fat content and volume<br>Pancreas fat content<br>Secondary diseases of diabetes mellitus type 2<br>Visceral and subcutaneous fat<br>Medical intake<br>Food intake<br>Quality of life<br>Sleep quality

Safety:

In the first six weeks, a low-calorie diet (800 kcal) will be used. The low-calorie diet is a protein-modified, fully balanced formula diet and it is in accordance with EU-Guidelines and the § 14 of the German Dietary Regulation. The formula diet consists of 70 g biological high-grade protein, 15 g fat for meeting the requirement in poly-unsaturated fatty acids and approximately 96 g carbohydrates. In addition, vitamins as well as macro- and micronutrients are added due to regulation. The EU-Guideline for Dietary Regulation from 05.05.1999 dictate a medical control during energy intake < 1000 kcal / day. This dictated medical control will be followed.

Study group and responsibilities

Study group

- Prof. Dr. M. Lerch (Department of Medicine A, University Medicine Greifswald)
- OÄ Dr. med. A. Steveling (Department of Medicine A, University Medicine Greifswald)
- M. Sc. oec. troph. L. Vogt (Department of Medicine A, University Medicine Greifswald)

Physician Investigator

- Dr. med. M. Kraft (Diakoniekrankenhaus Rotenburg an der Wümme)
- Dr. med. J.-P. Kühn (Institute of Radiology and Neuroradiology, University medicine Greifswald)
- Dr. med. A. Steveling (Department of Medicine A, University Medicine Greifswald)
- Prof. Dr. med. M. Masin (Department of Medicine B, University Medicine Münster)

Data and Safety Issues

- Dr. rer. nat. E. Weber (Department of Medicine A, University Medicine Greifswald)

Statistics

- Dr. Peter Meffert (Institute for Community Medicine / SHIP-KEF)

Contact

Prof. Dr. med. Markus M. Lerch
Department of Medicine A
Ferdinand-Sauerbruch-Str.
17475 Greifswald
Tel.: +49 3834/867230
E-Mail: lerch@uni-greifswald.de

Dr. med. Antje Steveling
Department of Medicine A
Ferdinand-Sauerbruch-Str.
17475 Greifswald

Tel.: +49 3834/866675
E-Mail: antje.steveling@uni-greifswald.de

Dr. med. Jens-Peter Kühn
Department of Radiology and Neuroradiology
Ferdinand-Sauerbruch-Str.
17475 Greifswald
Tel.: +49 3834/862170
E-Mail: kuehn@uni-greifswald.de

Dr. med. Matthias Kraft
Elise-Averdieck-Str. 20
27356 Rotenburg an der Wümme
Tel.: +49 4261/77-2090
E-Mail: kraftm@diako-online.de

Prof. Dr. Markus Masin
Department of Medicine B
University Medicine Münster
Albert-Schweitzer-Str. 33
48129 Münster

Dr. Peter Meffert
Institute for Community Medicine / SHIP-KEF
Walther-Rathenau-Str. 48
17475 Greifswald
Tel.: +49 3834/8619579

M. Sc. oec. troph. Lena J. Vogt
Department of Medicine A
Ferdinand-Sauerbruch-Str.
17475 Greifswald
Tel.: +49 3834/867268
E-Mail: lena.vogt@stud.uni-greifswald.de

Aims

In a dual center and prospective intervention study at the Department of Medicine A, University Medicine Greifswald and the Department of Medicine B, University Medicine Münster, the influence of a multimodal intervention therapy based on a standardized weight-loss program of parameters of metabolism in obese diabetics will be investigated.

Primary Endpoint

We estimated that the standardized weight-loss program effects a positive reduction of the HbA_{1c}-value about -15 % next to a successful weight loss. Therefore, HbA_{1c}-value is the primary endpoint.

Secondary Endpoint

- Parameters of diabetic metabolism:
    - Insulin sensitivity (HOMA index)
    - Serum concentration of total cholesterol, LDL- and HDL-cholesterol
    - Serum concentration of triglycerides, albumin, ALT, AST, GGT, creatinine, urea, C-peptide, proinsulin, IGF-1, growth hormone, sex-hormone-binding globulin, testosterone, thyroid-stimulating hormone, triiodothyronine, thyroxine
    - Liver fat content and volume (MRT)
    - Pancreas fat content and volume (MRT)
- Secondary diseases of diabetes mellitus type 2: microalbuminuria, diabetic foot syndrome, polyneuropathy, blood pressure
- Medical intake of oral antidiabetics and insulin
- Food intake (OptiDiet®)
- Visceral and subcutaneous fat (MRT/BIA)
- Quality of life (SF-12)
- Sleep quality (Pittsburgh sleep quality index)

Recruitment of the study population

After checking for in- and exclusion criteria through the study team, patients will be included in the trial. Every study patient will get an identification code, which will be saved at the University Medicine Greifswald. Blood samples and questionnaires will be collected. In addition, for every patient a registration sheet will be created with the following data: identification code, written informed consent, master data, diagnosis, and therapy.

Inclusion criteria

- BMI > 27 kg/m² and < 45 kg/m²
- Diabetes mellitus type 2
- Age > 18 and < 70 years
- Written informed consent
- HbA_{1c}–value and weight data for the last three month

Exclusion criteria

- Intake of incretin analogs ≤ 3 month before study inclusion
- Renal insufficiency or liver insufficiency (KDOQI > 3; Child Pugh > A)
- Heart failure (NYHA > 3)
- Diagnosed eating disorder
- Mental retardation
- Immobility
- Age < 18 years
- Pregnancy or breastfeeding
- Alcohol and drug abuse
- Exclusion from MRI-measurement: pacemaker, claustrophobia, metal-containing elements

The retrospective data for HbA_{1c}-value and weight will be used as controls.

Background

Obesity and Diabetes mellitus Type 2

Obesity is a pathological increase in body fat mass (BMI > 30 kg/m²) associated with health risks [1]. According to the "Nationale Verzehrsstudie 2" Germany (Second National Consumption Study), more than 68 % of men and about 50 % of women between the ages of 18 and 80 are overweight, of which 21 % and 20 % are obese [2]. At the same time, according to the data from the Federal Statistical Office and the Study of Health in Pomerania (SHIP-Study), in Pomerania are more obese adults (52,8%) compared to other federal states in Germany [3][4]. Due to these numbers as well as an increasing incidence and prevalence, obesity and the secondary diseases must be regarded as one of the biggest health problems in Germany. There is a marked increased risk of morbidity and mortality, which results in the indication of lifelong patient care [5].

Large prospective studies have shown that an increasing BMI is associated with a variety of secondary diseases as well as an increasing reduction in life expectancy [1][6]. The average life expectancy for obese persons at the age of 40 years without further risk factors (i.e. smoking) is reduced by seven years [7]. Not every obese person inevitably develops secondary diseases, but the risk increases with the extent and duration of obesity [1]. The major comorbidities and complications of obesity are: arterial hypertension, cardiovascular disease, type 2 diabetes mellitus, cancer and the development of fatty liver disease [8].

Obese persons have an up to 5-fold increased risk for developing diabetes mellitus [9][10]. The term diabetes mellitus encompasses a heterogeneous group of glucose metabolism disorders that are characterized by a pathological control of glucose metabolism with consecutive blood glucose elevation. This is caused by a lack of insulin or insufficient insulin action (insulin resistance). 80% of all diabetics have type 2 diabetes. The main causes are insulin resistance with a relatively insulin deficiency leading to a predominantly secretory defect with insulin resistance [11].

Insulin is a peptide hormone and is secreted in the pancreatic Langerhans' β cells. The insulin secretion induced by exocytosis of the β-granules is dependent on the level of glucose concentration in the extracellular space. If the glucose level rises above the fasting level of 4 mmol/l, insulin rapidly releases. Up to a glucose concentration of approximately 15 mmol/l, the amount of insulin released is proportional to the glucose concentration. In addition, branched-chain amino acids, gastrointestinal hormones and

drugs (i.e. sulfonylurea) induce insulin secretion. In contrast, catecholamine are important physiological inhibitors of insulin secretion. The insulin action is triggered by the insulin receptor binding, whereby the glucose uptake is stimulated by translocation of the glucose transporter GLUT-4 into the plasma membrane of the skeletal muscle and the liver. After absorption, the glucose is used for the synthesis of glycogen and thus for energy storage. At the same time, the increased glucose uptake leads to a decrease in the blood glucose concentration [12].

In obese persons often glucose intolerance occurs. That means, the glucose utilization in the muscle is reduced (peripheral insulin resistance) and an increased gluconeogenesis takes place in the liver (hepatic insulin resistance). In the early stages, an increased release of insulin with concomitant hyperinsulinemia compensated the insulin resistance. Over time, there is a risk of a decrease in the secretory function of the pancreatic β cells [13]. Inadequate treatment of diabetes may lead to late complications including cardiovascular disease, retinopathy, and diabetic nephropathy, diabetic neuropathy, amputations, and autonomic nervous system disorders with paresis in the gastrointestinal tract [14].

Professional societies initially recommend lifestyle interventions for obesity before medical or surgical therapies are considered [8][9]. Programs with comprehensive care by physicians, behavioral therapist, nutritionists and sport therapist, often in combination with formula diet as part of a structured weight-loss program, showed sufficient results in terms of sustainable weight loss [15][16][17]. Such intensively managed lifestyle interventions can also be successful in patients with diabetes mellitus type 2 and can reduce diabetes-associated mortality [15][18]. However, so far many research group investigated lifestyle interventions more for prevention than as therapy option [19][20][21]. Therefore, the OPTIFAST®II-Kurzprogram (short program) for obese patients with diabetes mellitus type 2 will be investigated at the Department of Internal Medicine A, University Medicine Greifswald and the Department of Medicine B, University Medicine Münster. Therefore, overweight and obese patients with diabetes mellitus type 2 get the chance to lose weight and to improve their metabolism. Additional survey will observe the insulin sensitivity and the liver and pancreas fat content during weight loss.

The OPTIFAST®-Program

In a one-week preparatory phase, a comprehensive health analysis will be conducted to check for the inclusion criteria and to exclude potential health risks. This includes laboratory analysis and cardiovascular risk assessment. In this phase, the analysis of the

previous nutritional behavior is carried out with the help of the methods established in die out-patient clinic (explanations in the Methods section).
This preparation phase is followed by a fasting period of six weeks. At the same time, an individual exercise program is worked out with the patient. The low-calorie diet is a protein-modified, fully balanced formula diet and it is in accordance with EU-Guidelines and the § 14 of the German Dietary Regulation. The formula diet consists of 70 g biological high-grade protein, 15 g fat for meeting the requirement in poly-unsaturated fatty acids and approximately 96 g carbohydrates. In addition, vitamins as well as macro- and micronutrients are added due to regulation. The EU-Guideline for Dietary Regulation from 05.05.1999 dictate a medical control during energy intake < 1000 kcal / day. This dictated medical control will be followed.
In the subsequent 4-week conversion phase, the gradual conversion to an energy-reduced balanced mixed diet according to the Guidelines of the German Society of Nutrition (Deutsche Gesellschaft für Ernährung, DGE) with a fat content of <30% and a carbohydrate content of >50-55%. Individual nutritional plans are created with the patient. At the same time, the exercise program will be expanded and deepened. By the 11th week begins the 5-week stabilization and intensification phase, in which the participants sustainably train and deepen the newly learned eating and exercise behavior. This phase is the heart of the program according to the experience, that the relapse of rigid patterns is the most prevalent at this time. Nutritional counseling and the psychological concept of flexible behavioral control with pyramid training are of particular importance.
During the 15 week-program, there will be a group training once a week with an average duration of 90 minutes. Trained nutritionists, dieticians, psychologist and physiotherapists give these trainings. The OPTIFAST® program sets the content for each week in a detailed, concrete therapy manual. Core topics include behavioral therapy, nutritional counseling, exercise, cooking classes, and medical focus training. In addition, it the manual contains worksheets, summaries and special videos that provide the basic information in an understandable way. Through these media, which every patient receives a high standardization of the content and statement is guaranteed. Through the written documents and communication with the group therapist in the sessions, step-by-step training assignments are agreed, which progress on a weekly basis. These therapeutic elements are based on psychologic learning and fulfill the requirements of a behavioral therapeutic intervention.
The aim of the program is a long-term weight stabilization by learning and training a healthy and active lifestyle. The conception of the therapy model fulfill all demands

according to the latest finding in the treatment of obesity: training in flexible behavioral control, breaking down rigid behavioral strategies, increasing the active movement in everyday life, realistic claim leveling as failure prophylaxis, social support through group dynamic processes, permanent medical, and psycho-psychological monitoring as well as long-term determination of the training program over 15-weeks with follow-up program.

Laboratory analyzes are routinely performed and documented at predetermined times during the program at weeks 0, 3, 7 and 13. In addition, all obesity relevant test results, especially the weight history, regularly documented to monitor the success or failure of therapy.

Methods

Nutritional status

Measurement of clinical relevant parameters

The measurements of the following parameters are standard routine in the hospital: glucose, insulin, HbA_{1c}, triglyceride, HDL-cholesterol, LDL-cholesterol, total cholesterol, ALT, AST, GGT, AP, creatinine, urea, bilirubin, thyroid-stimulating hormone, triiodothyronine, thyroxine, small blood count, sodium, potassium, calcium, 25- und 1,25-vitamine D, phosphate, uric acid, sex-hormone-binding globulin, testosterone, IGF-1, lutein, prolactin.

The blood samples determining DNA- and RNA-expression as well as stool sample for DNA-analysis will be analyzed and stored by the research laboratory of Department of Medicine A of University Medicine Greifswald.

Anthropometric data

During the intervention period, patients will be weighted weekly. Body mass index is calculated as body weight divided by height squared [kg/m^2]. In addition, waist and hip circumference will be measured midway between the superior iliac spine and the lower rib margin without exerting pressure on the body surface.

Bioelectric impedance analysis

Bioelectric impedance analysis is used for determination of body composition of patients in week 1, 7 and 15. It is a widely used and non-invasive method for body composition and nutritional status based on electrical conductivity of the different body tissues. Technically two electrodes are placed on the patient`s body surface and a weak electric field is be generated that is not felt by the patient. There are two types of electric resistances that are determined; one is water resistance (R out of which body fluid content, lean body mass and fat will be determined. Cellular resistance (Xc) is the second parameter and gives information on organ and muscular mass of the body. Final body composition is calculated by Data Input® [22].

OptiDiet

To compare food intake of the patients before and after the intervention, patients will complete a nutritional protocol with all food and beverages they consumed in seven days. To calculate the average intake, the software OptiDiet® will be used. OptiDiet® gives information and recommendations on a variety of diets and allergies, and contains data on nutritional values and nutrients. OptiDiet® is used for preparation of nutrition protocols and detailed calculations of nutritional components. Recommendations are based on the guidelines of the German Society for Nutrition (Deutsche Gesellschaft für Ernährung, DGE), "Rationalisierungsschema" (concept of different diets for clinical nutrition) of the German Society of Nutrition Medicine (Deutsche Gesellschaft für Ernährungsmedizin, DGEM) and several books about nutrition medicine and dietetics.

HOMA-Index

The homeostasis-model-assessment-(HOMA)-index will be used for calculating insulin sensitivity. For calculation, fasting-insulin and fasting-glucose-values are necessary. The HOMA index reflects the glucose-insulin-homeostasis. The cut-off value for insulin resistance in adult patients is > 2.5. The calculation is based on the formula [23]: fasting-insulin [µU/ml] multiplied by fasting-glucose [mmol/l] divided by 22.5.

Magnetic resonance imaging

Magnetic resonance imaging (MRT 1.5 Tesla Magnetom Avanto, Siemens HealthCare, Erlangen) is a valid tool for fat quantification (liver fat, visceral fat, pancreas fat). There is a standardized protocol including allocation for the right position, adjustment and calibration. One measurement will last 10 minutes. The MRI is a diagnostic assessment tool without using ionizing ration (X ray). Instead, airwaves and magnetic fields were used. No contrast media is given to the patient. All contraindication will be checked before the first measurement using an additional clinical accepted information sheet.

Quality of Life

The questionnaire SF-12 will be used for the measurement of quality of life. The SF-12 is the short form of the measurement tool SF-36 Health Survey. Both, the general health status and the impairment in every-day work will be assessed. The results are presented

as scale values between 0 and 100. A lower score equals a lower quality of life.

Sleep quality

The sleep quality before and after the intervention will be assessed using the Pittsburgh sleep quality index (PSQI). The questionnaire conducted retrospectively for the last four weeks the amount of sleep-disturbing events, the estimation of the sleep quality, the general sleeping time, latency of falling asleep and sleep duration, usage of sleep medication, tiredness during the day. The test consist of seven components, of which each can have a value between 0 and 3. The values were summarized to the general sleep quality. A higher value equals a poorer sleep quality.

Many research groups investigated the diagnostic validity including sensitivity and specificity. The sensitivity was over 80% (80-100%), the specificity (only analyzed in three trials) showed equally high values (83-87%) [24].

Study design

Initiation of the study

After approval of the study by the local ethics committee recruitment could be started. In addition, this study will be registered at the database ClinicalTrials.gov (NCT).

Study procedure

Figure 1 shows the study procedure. Before the weight-loss program started, patients will be properly informed and after written informed consent, the baseline measurements will be collected. The baseline measurements will include: MRI- and BIA-measurements, blood samples, anthropometric data, 7-day food-diary, questionnaire on quality of life and sleep quality. These measurements will be repeated after 15 weeks weight-loss program.
The weight-loss program will last 15 weeks and consisted of the OPTIFAST®-Kurzprogram (short program) and additional measurements. Weight, waist and hip circumference will be measured weekly. An additional MRI- and BIA-measurement will be conducted in week 7. Two additional blood samples will be conducted for patients` safety. The necessary medical care will be ensured for the whole study period.
For assessing long-term effects, two follow-up measurements are planned after three, six and nine month. In case of weight gain, patients will have the possibility to receive a food replacement therapy.

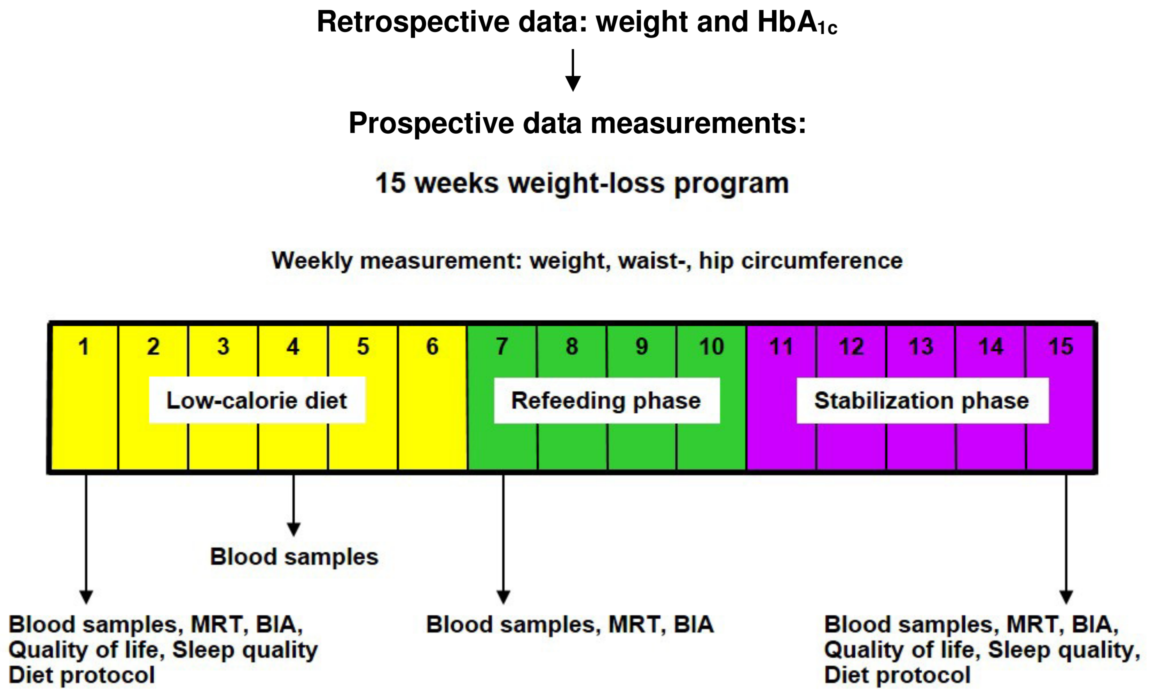

Figure 1: Time points for data measurements

Study termination

The study will be terminated in presence of the following factors:

- Patient`s wish: the patient can withdraw from the study at any time he/she wants.
- If the therapy is disadvantageous for the patient.

Any deviation or early termination of the study has to be recorded by a member of the study group. If possible, examinations that were originally scheduled at the end of the study should be brought forward. The patient has the right to withdraw from the study at any time. Any adverse event will have to be recorded and the patient observed until clarification of the adverse event.

Financial support

Nestlé Health Science Germany support the study by granting study participants a 15 % discount for the formula diet.

Statistical considerations

The retrospective data of HbA_{1c}-values before the intervention will be used as controls for the paired sample. Die changes before intervention will be compared with changes during the intervention Therefore, Wilcoxon signed-rank test will be used. In case of normal distribution, the t-test can be used also. Based on recent literature, sample size calculation resulted in n = 35 with a power of 80% and n = 57 with a power of 95% (Figure 2). Statistical analysis will be done using STATA 11 software.

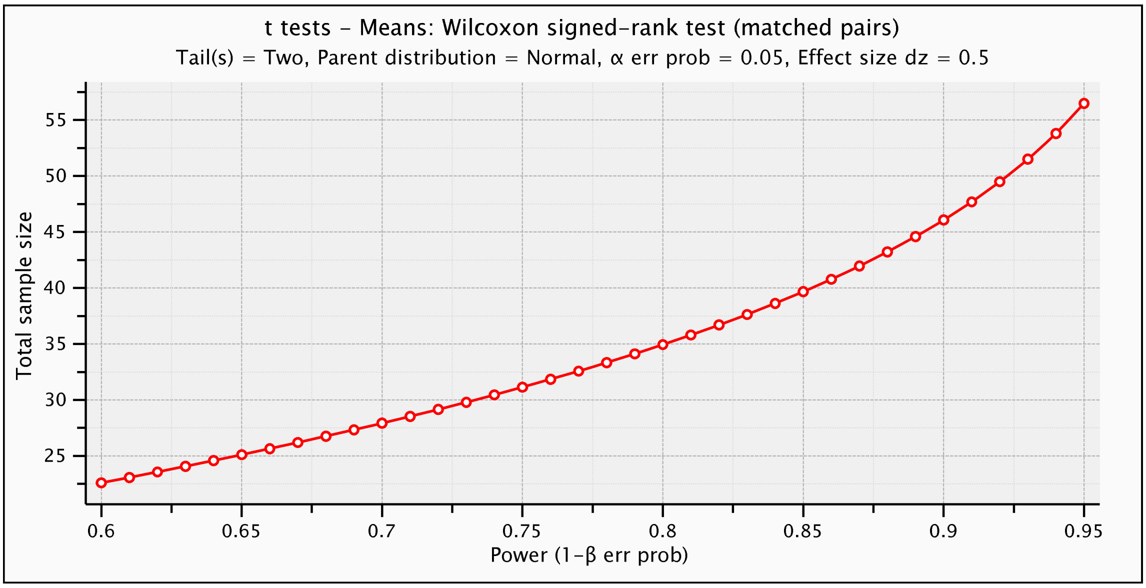

Figure 2: Relation of sample size and power

Ethical and data safety issues

Data will be saved electronically in a pseudonymized way using an identification code. All data will be stored on one computer belonging to the Department of Medicine A, University Medicine Greifswald. Only members of the study group are authorized to have access to pseudonymized data.

References

1. Hauner H: Adipositas – Klinik und Ernährungstherapie: Schauder P und Ollenschläger G: Ernährungsmedizin – Prävention und Therapie, Urban und Fischer, 2. Auflage: 537-567

2. Bundesministerium für Ernährung, Landwirtschaft und Verbraucherschutz und Max Rubner Institut, Bundesforschungsinstitut für Ernährung und Lebensmittel, Standort Karlsruhe: Nationale Verzehrsstudie II (2005-2009), Stand: 25.05.2011

3. Puls R, Völzke H: Ganzkörper-MRT in der „Study of Health in Pomerania“. Radiologe, 2011; 51:379-383

4. Statistisches Bundesamt, Wiebaden: Körpermaße der Bevölkerung. Mikrozensus – Fragen zur Gesundheit, 2009

5. Aucott L, Gray D, Rothnie H, Thapa M, Waweru C: Effects of lifestyle interventions and long-term weight loss on lipid outcomes – a systematic review. Obesity, 2011; e412-e425

6. Fontaine KR, Redden DT, Wang C, Westfall AO, Allison DB: Years of life lost due to obesity. JAMA, 2003; 289:187-193

7. Haslam DW, James PT: Obesity. Lancet, 2005; 366:1197-209

8. Hauner H, Buchholz G, Hamann A, Husemann B, Koletzko B, Liebermeister H, Wabitsch M, Westenhöfer J, Wirth A, Wolfram G: Prävention und Therapie der Adipositas – Evidenzbasierte Leitlinie. 2007; 1-29

9. Alberti KGMM, Eckel RH, Grundy SM, Zimmet PZ, Cleeman JI, Donato KA, Fruchart JC, James WPTJ, Loria CM, Smith SC: Harmonizing the Metabolic Syndrome: A joint Interim Statement of the International Diabetes Federation Task Force on Epidemiology and Prevention; National Heart, Lung and Blood Institut; American Heart Association and International Association for the Study of Obesity. Circulation, 2009; 120:1640-1645

10. Mack F, Abeygunawardhana N, Mundt T, Schwahn C, Proff P, Spassov A, Kocher T, Biffar R: The factors associated with body mass index in adults from the study of health in Pomerania (SHIP-0), Germany. Journal of Physiology and Pharmacology, 2008; 59:5-16

11. Toeller M: Diabetes mellitus: Schauder P und Ollenschläger G: Ernährungsmedizin – Prävention und Therapie, Urban und Fischer, 2003, 2. Auflage: 713-722

12. Löffler G: Regulation des Intermediärstoffwechsels. Löffler G: Basiswissen Biochemie mit Pathobiochemie, Springer Medizin Verlag, 2005; 6. Auflage:350-356

13. Burkhard M: Adipositas und metabolisches Syndrom. Koula-Jenik H, Kraft M, Miko M, Schulz RJ: Leitfaden Ernährungsmedizin, Urban und Fischer, 2006, 1. Auflage:419-436

14. Gellner R, Hengst K. Diabetes mellitus und Folgeerkrankungen. Koula-Jenik H, Kraft M, Miko M, Schulz RJ: Leitfaden Ernährungsmedizin, Urban und Fischer, 2006, 1. Auflage:455-468

15. Bischoff SC, Damms-Machado A, Betz C, Herpertz S, Legenbauer T, Löw T, JG Wechsler, Bischoff G, Austel A, Ellrott T: Multicenter evaluation of an interdisciplinary 52-week weight loss program for obesity with regard to body weight, comorbidities and quality of life - a prospective study. International Journal of Obesity, 2011

16. Wadden TA, Frey DL: A Multicenter Evaluation of a Proprietary Weight Loss Programm for the Treatmend of Marked Obesity: a five-year follow-up. Int J Eat Disord, 1997; 22:203-212

17. Norris SL, Zhang X, Avenell A, Gregg E, Brown T, Schmid CH, Lau J: Long-term non pharmacological weight loss interventions for adults with type 2 diabetes mellitus . 2005

18. Williamson DF, Thompson TJ, Thun M, Flanders D, Pamuk E, Byers T: Intentional Weight Loss and Mortality among Overweight Individuals with Diabetes. Diabetes Care, 2000; 23:1499-1504

19. Reduction in the incidence of type 2 diabetes with lifestyle intervention or metformin. N Engl J Med, 2002; 346:393-403

20. :Lindström J, Louheranta A, Mannelin M, Rastas M, Salminen V, Eriksson J, Uusitupa M, Toumilehto J. The finnish diabetes prevention study (DPS). Diabetes Care, 2003; 26:3230-3236

21. Lindström J, Eriksson JG, Valle TT, Aunola S, Cepaitis Z, Hakumäki M, Hämäläinen H, Ilanne-Parikka P, Keinännen-Kiukaanniemi S, Laakso M, Louheranta A, Mannelin M, Martikkala V, Moltchanov V, Rastas M, Salminen V, Sundvall, Uusitupa M, Tuomilehto J: Prevention of diabetes mellitus in subjects with impaired glucose tolerance in the finnish diabetes prevention study: results from a randomized clinical trial. J Am Soc Nephrol, 2003; 14:S108-S113

22. Kushner RF, Kunigk A, Alspaugh M, Andronis PT, Leitch CA, Schoeller DA: Validation of bioelectrical-impedance analysis as a measurement of change in body composition in obesity. Am J Clin Nutr, 1990; 52:219-23

23. Matthews DR, Hosker JP, Rudenski AS, Naylor BA, Treacher DF, Turner RC: Homeostasis model assessment: insulin resistance and beta-cell function from fasting plasma glucose and insulin concentrations in man. Diabetologia, 1985; 28:412-419

24. Buysse DJ, Reynolds III CF, Monk TH, Berman SR, Kupfer DJ. The Pittsburgh Sleep Quality Index: A new instrument for psychiatric practice and research. Psychiatry Research 1989, 28:193-213
